# Clinical features, pathogenesis, and treatment of myasthenia gravis: a supplement to the Guidelines of the German Neurological Society

**DOI:** 10.1007/s00415-016-8045-z

**Published:** 2016-02-17

**Authors:** Nico Melzer, Tobias Ruck, Peter Fuhr, Ralf Gold, Reinhard Hohlfeld, Alexander Marx, Arthur Melms, Björn Tackenberg, Berthold Schalke, Christiane Schneider-Gold, Fritz Zimprich, Sven G. Meuth, Heinz Wiendl

**Affiliations:** 1 Department of Neurology, University of Münster, Albert-Schweitzer-Campus 1, 48149 Münster, Germany; 2 Department of Neurology, University of Basel, Basel, Switzerland; 3 Department of Neurology, University of Bochum, Bochum, Germany; 4 Institute of Clinical Neuroimmunology, Ludwig-Maximilians-University Munich, Munich, Germany; 5 Institute of Pathology, University Medical Centre Mannheim, University of Heidelberg, Mannheim, Germany; 6 Department of Neurology, University of Erlangen, Erlangen, Germany; 7 Department of Neurology, University of Marburg, Marburg, Germany; 8 Department of Neurology, University of Regensburg, Regensburg, Germany; 9 Department of Neurology, Medical University of Vienna, Vienna, Austria

**Keywords:** Myasthenia gravis, Pathogenesis, Treatment guidelines

## Abstract

Myasthenia gravis (MG) is an autoimmune antibody-mediated disorder of neuromuscular synaptic transmission. The clinical hallmark of MG consists of fluctuating fatigability and weakness affecting ocular, bulbar and (proximal) limb skeletal muscle groups. MG may either occur as an autoimmune disease with distinct immunogenetic characteristics or as a paraneoplastic syndrome associated with tumors of the thymus. Impairment of central thymic and peripheral self-tolerance mechanisms in both cases is thought to favor an autoimmune CD4^+^ T cell-mediated B cell activation and synthesis of pathogenic high-affinity autoantibodies of either the IgG1 and 3 or IgG4 subclass. These autoantibodies bind to the nicotinic acetylcholine receptor (AchR) itself, or muscle-specific tyrosine-kinase (MuSK), lipoprotein receptor-related protein 4 (LRP4) and agrin involved in clustering of AchRs within the postsynaptic membrane and structural maintenance of the neuromuscular synapse. This results in disturbance of neuromuscular transmission and thus clinical manifestation of the disease. Emphasizing evidence from clinical trials, we provide an updated overview on immunopathogenesis, and derived current and future treatment strategies for MG divided into: (a) symptomatic treatments facilitating neuromuscular transmission, (b) antibody-depleting treatments, and (c) immunotherapeutic treatment strategies.

## Introduction

Myasthenia gravis (MG) is regarded an autoimmune antibody-mediated disorder of neuromuscular synaptic transmission as (a) auto-antibody depositions are detectable at the neuromuscular junction (NMJ) [175, 176]; (b) autoantibodies from MG patients cause MG symptoms when passively transferred into rodents [175, 176]; (c) active immunization of animals with auto-antigens reproduces the disease [59]; and (d) antibody-depleting therapies decrease the severity of MG symptoms [43, 120, 127].

The incidence of MG ranges from 0.25 to 2.0 per 1,000,000. Due to effective treatment strategies and normal life expectancy, the prevalence of MG has risen in recent years to about 72:1,000,000 (range 15–179 [15]). About 10 % of patients are children and adolescents. There is an increased familial risk for MG. Siblings or first-grade relatives of affected patients have a risk of 4.5 % for developing MG reflecting a profound genetic disposition for the disorder [71].

The clinical hallmark of MG consists of fluctuating fatigability and weakness affecting ocular, bulbar and (proximal) limb skeletal muscle groups. A pragmatic clinical classification distinguishes pure ocular myasthenia from generalized myasthenia with mild, moderate and severe manifestation. Ocular myasthenia exclusively affects the outer ocular muscles including the M. levator palpebrae and presents with ptosis and double vision. Ptosis and double vision may be transient, fluctuating or progressive during the day. Only 10–20 % of patients show muscle fatigability and weakness persistently restricted to the outer ocular muscles. The majority of patients proceed to generalized muscle fatigability and weakness within 24 months after the disease onset [135]. Generalized myasthenia is defined as any clinical affection of muscle groups other than outer ocular muscles independent of its severity.

The fluctuating muscle fatigability and weakness is illustrated by a typical decremental response of the amplitude and/or area under the curve of the elicited muscle compound action potential of the fifth compared to the first stimulus upon repetitive supramaximal stimulation of the accessory or facial nerve with a frequency of 3 Hz before and after isometric tetanic contraction [27]. The lack of an incremental response of amplitudes and areas under the curve of the compound muscle action potential upon supramaximal repetitive nerve stimulation using a frequency of 30 Hz or upon pre- and post-tetanic single stimulation proofs the post-synaptic nature of the neuromuscular transmission defect [27]. Single fiber electromyography typically shows increased jitter and intermittent conduction blocks [145, 179] reflecting instable neuromuscular transmission.

## Epidemiological, immunological, and genetic features of distinct MG subtypes

Based on clinical, epidemiological, immunological and genetic [60, 134, 151] findings as well as thymus pathology, MG has been further sub-classified (Table 1): pure ocular MG (OMG; [135, 169]) is distinguished from generalized MG with early onset (<45 years “early-onset” MG, EOMG) and generalized MG with late onset (>45 years “late-onset” MG, LOMG). EOMG is often associated with lymphofollicular hyperplasia of the thymus, and LOMG is characterized by age-dependent involution of the thymus. In contrast, 10–15 % of all patients do have thymoma (thymoma-associated MG, TAMG).

**Table 1:** Features of different subtypes of MG

	Early-onset MG (EOMG)	Late-onset MG (LOMG)	Thymoma-associated MG (TAMG)	Anti-MuSK-Ab-associated MG (MAMG)	Ocular MG (OMG)	“Seronegative”MG (SNMG)
Estimated frequency	20 %	45 %	10–15 %	6 %	15 %	4 %
Disease-course and manifestation	Generalized, disease maximum within the first 3 years	Generalized, disease maximum within the first 3 years	Generalized, more scarcely—complete remission is possible	Generalized, fasciopharyngeal focus	Ocular	Generalized
Age at disease onset	≤45 (50, 60) years^a^	>45 (50, 60) years^a^	Any age (primarily 40–60 years)	Any age (primarily Younger patients)	Any age	Any age
Male: female ratio	1:3	5:1	1:1	1:3	1:2	n. a.
HLA-association (caucasians)	B8 A1 DR3 (strong) DR16 DR9 (less strong)	B7 DR2 (less strong) Anti-titin-ab^−^ with DR7 Anti-titin-ab^+^ with DR3	DR7 (less strong) A25 (less strong)	DR14 (strong)	n. a.	n. a.
(Auto-)antibodies	**Anti-AChR-ab**	**Anti-AChR-ab** Anti-Titin-ab Anti-RyR-ab Anti-IL12-ab Anti-IFNα-ab	**Anti-AChR-ab** Anti-Titin-ab Anti-RyR-ab Anti-IL12-ab Anti-IFNα-ab Anti-IFN*ω*-ab	**Anti-MuSK-ab**	**Anti-AChR-ab** (50–70 %)	**Anti-LRP4-ab** **Anti-Argin-ab**
Typical thymus pathology	lymphofollicular hyperplasia (LFH)	Atrophy, involution	Thymoma Type A 5 % Type AB, B1–3 92 %	Usually normal	No systematic data	No systemic data
Response to thymectomy	good when performed within the first 1–2 years after diagnosis	No systematic data	Often poor	Poor	No systematic data	No systematic data
Response to immunotherapy	+++	+++	+(+)	+(+)	+++	+(+)

*n. a.* not applicable

^a^Currently there is no agreement in the literature regarding the age differentiating EOMG from LOMG [20, 104]

MG is due to a reduction of functional skeletal muscle nicotinic acetylcholine receptors (AChR) at and structural alterations of the neuromuscular endplate due to the effects of different autoantibodies. In about 85 %, autoantibodies against the AChR itself can be detected. The AChR is a pentameric ligand-gated monovalent cation channel that exists in two forms with defined stoichiometry of the homologous alpha (α), beta (β), gamma (γ), delta (δ) and epsilon (ε) subunits: the fetal AChR shows an α_2_βδγ subunit composition, and the adult AChR shows an α_2_βδε subunit stoichiometry. The α-subunit contains two functionally important domains: (a) an extracellular cystein loop that mediates ligand (acetylcholine, ACh) binding [1]; and (b) an extracellular sequence to which most AChR autoantibodies are binding termed the main immunogenic region (MIR) [102, 181].

During development and following muscle innervation the γ-subunit of the fetal AChR is replaced by the ε-subunit yielding adult AChRs [146]. Normally, only skeletal muscle cells and thymic myoid cells express functional AChRs consisting of folded subunits [185]. In the normal thymus, both adult and fetal AChR are expressed by non-innervated thymic myoid cells that likely play a role in the induction of central immunological tolerance towards muscle proteins [148].

In addition, unfolded AChR subunits (but not whole functional channels [157]) are expressed by thymic epithelial cells [128], partly under the control of the autoimmune regulator (AIRE) [55]. AIRE regulates the presentation of AChR peptides by MHC molecules to developing T cells and normally supports immunological tolerance towards the AChR.

**Fig. 1:**
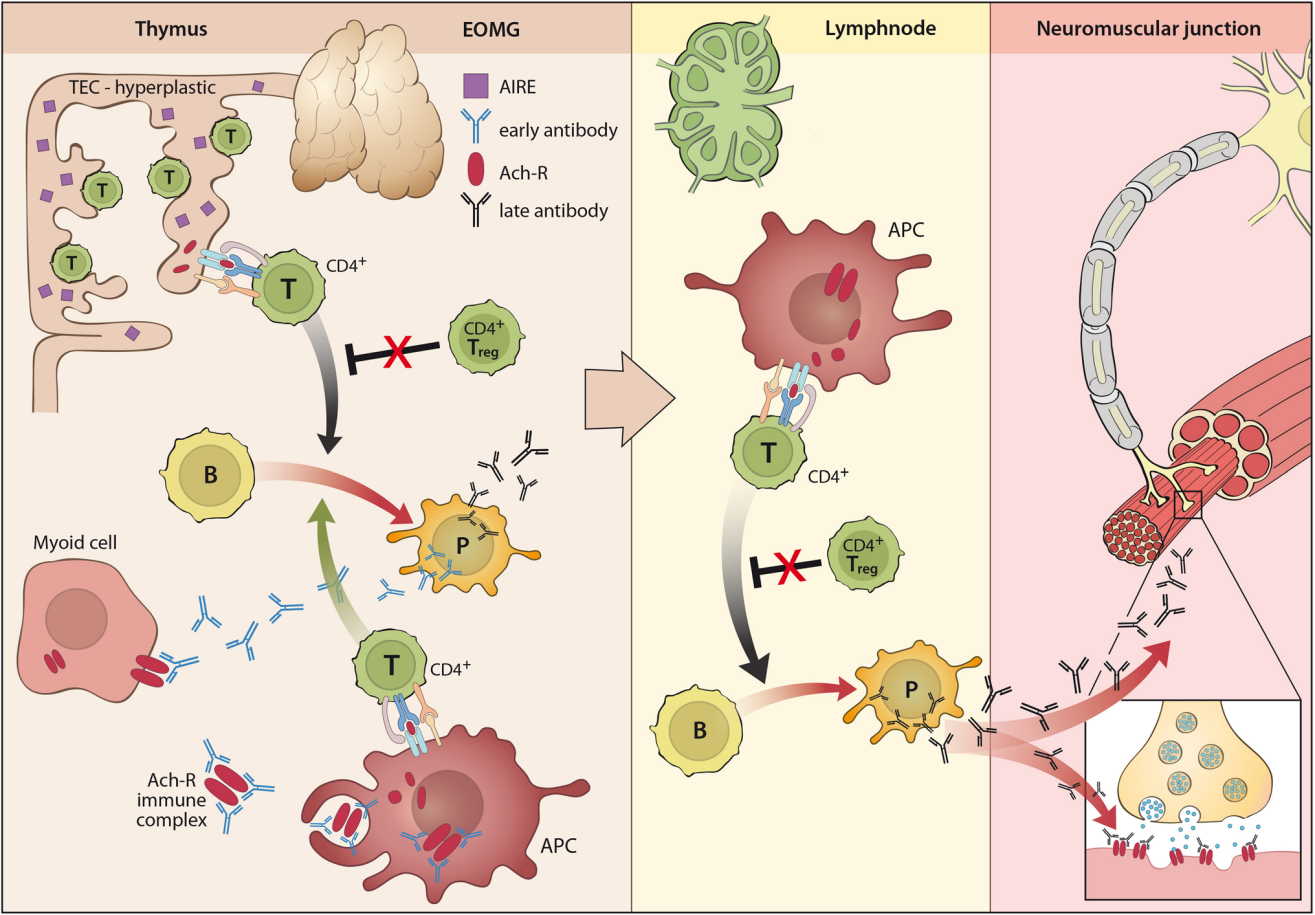
Pathogenesis of early-onset MG (EOMG) with lymphofollicular hyperplasia (LFH)

**Fig. 2:**
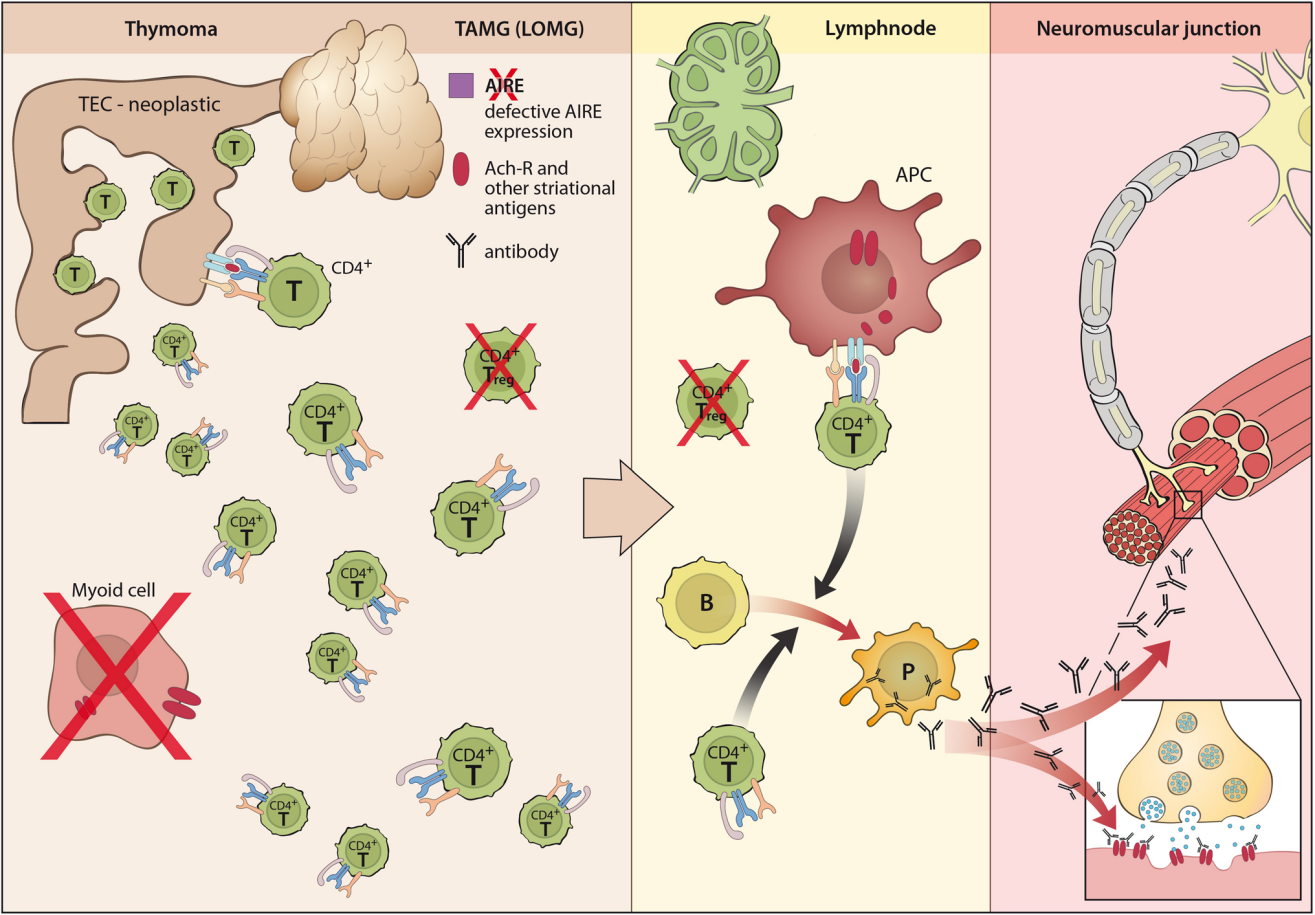
Pathogenesis of thymoma-associated (and late-onset) MG (TAMG, LOMG)

Antibodies against AChR are in part low affinity antibodies (5 %) that in contrast to high affinity antibodies (80 %) can only be detected as clusters on the cell surface in cell-based assays (CBA) but not in solubilized from in standard radioimmunoassays (RIA) [98]. Levels of antibodies against the “main immunogenic region” (MIR) of the AChR are of the complement-binding IgG1 and three type and correlate with disease severity [109]. These antibodies (Figs. 1, 2) may (a) block the receptor and (b) lead to its internalization reducing the number of available receptors in the membrane. Moreover, (c) activation of the complement cascade leads to destruction of the endplate architecture with a widened synaptic cleft (i.e., the distance between the pre-synaptic acetylcholine release site and the post-synaptic endplate), increasing the distance for acetylcholine molecules to diffuse from their release sites to their receptors [109].

Upon the initiation, the humoral AChR-directed autoimmune response in MG is usually focused on single epitopes of the α-subunit of the AChR. However, during the disease course, the focus may spread also to other epitopes within the α-subunit or even other subunits or antigens [184] due to secondary involvement (i.e., professional processing and presentation) of natural AChRs derived from muscle or thymic myoid cells [28, 75, 103].

Antibodies against muscle-specific tyrosine kinase (MuSK) are of the non-complement binding IgG4 type and prevent the interaction of low-density lipoprotein receptor-related protein 4 (LRP4) with MuSK disturbing the agrin-induced architecture of the neuromuscular junction [23, 77, 85, 92, 110, 155, 156]. Antibodies against LRP4 are predominantly of the complement-binding IgG1 and 2 type and are able to inhibit the LRP4-agrin interaction and thus alter AChR clustering in muscle cells [9, 74, 125, 154, 196, 199]. Antibodies against agrin are able to inhibit agrin-induced MuSK phosphorylation and AChR clustering in muscle cells [51, 195]. The IgG subtype classification of antibodies against agrin has not yet been studied [51, 195].

AChR and MuSK antibodies usually do not occur together in a single patient [33] whereas overlap of LRP4 antibodies with both AChR and MuSK antibodies in individual patients has been reported [199]. Moreover, agrin antibodies have been also detected in combination with antibodies against MuSK, LRP4, or AChR, indicating a high incidence of autoantibodies against several neuromuscular proteins in the agrin-positive MG cases [51, 195].

## Role of the thymus in distinct MG subtypes

MG may either manifest as an autoimmune disease with distinct immunogenetic characteristics or as a paraneoplastic syndrome associated with tumors of the thymus but only rarely with other malignancies [17, 106]. The thymus exhibits pathological changes in the majority of patients with AChR antibodies (most patients with OMG, EOMG, LOMG and TAMG; Table 1), which seem to be of central importance for the impairment of central and peripheral tolerance and initiation of immunopathogenesis of MG (Figs. 1, 2) [106]. Pathological changes of the thymus have also been reported in some patients with LRP4 antibodies [199]. However, thymoma and other thymic pathologies are rarely associated with MuSK antibody MG [97, 99], and data on thymus alterations in agrin antibody MG have not yet been described [51, 195].

Almost complete elimination of autoreactive T cells is usually achieved via interactions between thymic stromal cells (epithelial cells, dendritic cells and myoid cells) expressing or presenting self-antigens and developing thymocytes. Self-tolerant T cells continue their differentiation and finally become exported to the periphery. Under physiological conditions, the thymus contains mostly thymocytes (i.e., developing T cells) and stromal cells, and the number of B cells is very small [106].

About 70 % of patients with EOMG (Fig. 1) show lymphofollicular hyperplasia (LFH), i.e., thymitis with lymphoid follicles and germinal centers within the thymus [106]. Following an unknown initial “trigger”, hyperplastic, MHC-class II expressing thymic epithelial cells (TECs) seem to present unfolded AChR subunits and activate auto-reactive CD4^+^ T cells. Early antibodies elicited by primed T cells are supposed to attack nearby myoid cells expressing folded AChRs and activate complement with subsequent release of AChR/immune complexes. These AChR/immune complexes in turn activate professional antigen presenting cells that drive further activation of auto-reactive CD4^+^ T cells leading to further activation and expansion of auto-reactive B cells with affinity maturation of their B cell receptors leading to the production of high-affinity late AChR-antibodies and subsequent epitope diversification [186].

The intrathymic autoimmune inflammatory process in EOMG seems to be self-perpetuating likely due to dysfunctional regulatory T cells that have been described in the EOMG thymus and blood [8, 173] Apparently, the autoimmune process that is initiated in the thymus can later spread to the peripheral lymphatic tissue, where skeletal muscle-derived AChR/immune complexes in regional lymph nodes and functionally defective regulatory T cells may contribute to the maintenance of EOMG [106].

10–15 % of MG patients have a thymoma and about 30 % of thymoma patients have TAMG (Fig. 2). Thymomas are neoplasms of thymic epithelial cells (TECs) usually with mixed cortical and medullary properties [187]. According to lymphocyte content and epithelial cell features the current histological classification distinguishes type A, AB, B1, B2, and B3 thymomas [178]. More than 95 % of all thymomas (except for rare type A and B3) generate polyclonal CD4^+^ and CD8^+^ thymocytes from bone marrow progenitors [178]. Such thymopoiesis plays a central role in the pathogenesis of TAMG: MG-positive but not MG-negative thymomas generate and export large numbers of mature CD4^+^ CD45RA^+^ cells to the blood [14, 79, 164]. Accordingly, thymopoietically incompetent thymic carcinomas are not associated with MG [162].

However, active thymopoiesis in thymomas occurs under conditions favoring autoimmunity: thymomas exhibit defective expression of the autoimmune regulator AIRE [166] that normally drives ‘promiscuous’ expression of peripheral tissue autoantigens (including the AChR α-subunit [55]) in thymic epithelial cells, and thymomas have reduced or absent thymic myoid cells [108]. Moreover, neoplastic epithelial cells variably express striational antigen epitopes [29], including epitopes of titin [107, 138] and various AChR subunits (but not whole receptors) [157] together with reduced levels of MHC-class II [147, 163, 187]. These altered properties of neoplastic epithelial cells may profoundly interfere with (positive and negative) selection of maturing thymocytes and the activation status of mature T cells [103, 138]. Moreover, in concert with reduced levels of AIRE [3], these alterations result in defective generation of regulatory T cells by thymomas [167]. Together, these alterations of the thymic microenvironment favor the export of substantial numbers of naïve and pre-primed auto-reactive T cells into the periphery, which are expected to gradually replace the patient’s native, more tolerant T cell repertoire in the periphery [14, 79]. In the peripheral lymphatic tissue, they apparently stimulate the pathogenic B cell response after appropriate activation. This usually happens before, but rarely also after thymoma resection [164]. This impact of the thymoma on the peripheral immune system explains why TAMG, once initiated, is self-sustaining even after complete thymoma removal (that is typically accompanied by resection of the residual thymus). Again skeletal muscle-derived AChR/autoantibody complexes processed in the absence of regulatory T cells in regional lymph nodes might perpetuate TAMG.

While the autoimmune focus of EOMG is largely on the AChR, the spectrum of autoantibody targets in individual patients with TAMG can be much broader (Table 1) [108]. The spectrum of autoantigens may include: (a) Ligand- and voltage-gated ion channels, including the skeletal muscle AChR and, much rarer, voltage-gated Ca^2+^ and K^+^ channels and other ligand-gated neurotransmitter receptors, or proteins complexed with them. (b) Striational antigens with titin and ryanodin receptors (RyR) being the major autoantibody targets. (c) Cytokines including interferon-α (IFN-α), interferon-ω (IFN-ω), and interleukin-12 (IL-12). This extended spectrum of autoantigens in TAMG also explains the much more frequent occurrence of other autoimmune disorders in addition to MG in these patients.

Patients with LOMG (Fig. 2) show involution and atrophy of the thymus. The lympho-epithelial tissue of the normal aging thymus is gradually replaced by fat, but residual parenchyma may continue to export some T cells at least into adulthood [34]. In LOMG, this residual lymphoepthelial tissue may rarely show signs of expansion and even infiltration, however, morphometric analysis did not reveal significant differences between thymuses of patients with LOMG and normal thymuses [165]. Thymic myoid cells tend to be sparse in LOMG [90, 182], decline with age and can reach a state of near-deficiency between the age of 60 and 70 years, with considerable interpersonal variation [139]. In addition, the number of AIRE positive cells seems to decline as well, however, with no clear difference between LOMG thymuses and age matched controls.

LOMG patients show striking immunological parallels with TAMG (Table 1): (a) autoantibodies against titin occur in 70 % of patients (especially in those aged over 60 years) [14], and others have antibodies against RyR [137]. (b) Up to 40 % have neutralizing antibodies against IFNα and/or IL-12 [65, 91]. (c) More than 50 % share common expansions in the peripheral T cell repertoire with TAMG patients [79, 170, 171]. Hence, immunological parallels between LOMG and TAMG are so close that it appears that aberrations in the aged thymus in LOMG mimic thymoma behavior without definite neoplasia, leading to export and possibly even activation of non-tolerant T cells [108, 186]. Substantially increased export of naïve T cells has not been observed in LOMG patients at the time of diagnosis [14, 85]. However, a small thymoma could have regressed spontaneously before the diagnosis of MG [186]. Moreover, a small population of highly potent, AChR and titin reactive T cells generated in the near absence of myoid cells inside a largely AIRE-negative atrophic thymus could become activated after export to the periphery and trigger LOMG, and a pathogenic T cell population derived from an atrophic, myoid cell-poor and AIRE-negative thymus may have accumulated in the periphery over a long period before the outbreak of LOMG, i.e., similar to rare thymoma patients who develop TAMG years after thymoma removal [164]. Once initiated, LOMG could become self-perpetuating as described above for EOMG and TAMG, i.e., by stimulatory AChR/autoantibody complexes in muscle-draining lymph nodes.

## Treatment strategies for MG

Largely independent from the autoantibody status, MG is treated according to the same principles. Treatment strategies can be distinguished into (a) symptomatic treatments facilitating neuromuscular transmission and (b) immunosuppressive treatments targeting the underlying pathological immune response in MG.

### Symptomatic treatments

Acetylcholinesterase inhibitors (AChEI) such as pyridostigmin bromide represent the most common symptomatic treatments. Clinical efficacy of these drugs has been underlined using electrophysiological measurements. However, their broad application in the treatment of MG is based on uncontrolled observational studies, case series as well as on good clinical practice [111, 158]. For ethical reasons placebo-controlled studies on the clinical efficacy of these compounds are prohibited.

Patients with MuSK-antibody-associated MG usually respond worse than those with nAChR-antibody-associated MG to treatment with AChEI. In these cases, higher dosages are required for symptom control often leading to increased systemic side effects [40, 132].

Pyridostigmin bromide is nowadays used for the oral long-term treatment of MG. Cholinergic side effects usually do not occur at dosages below 300 mg/day. However, during intravenous application cholinergic side effects such as bronchial spasm and hypersecretion, aggravated myasthenic muscle weakness, abdominal crampi, urinary urgency, hypersalivation and sweating, bradycardia and arterio-ventricular block, and miosis may occur and constitute cholinergic intoxication. Intravenous application of pyridostigmin bromide (up to 24 mg/day) always requires continuous monitoring in an intermediate or intensive care setting. The most frequent systemic side effects during all modes of application include gastrointestinal disturbance, diarrhea and crampi (in about 30 %), hypersalivation (in about 6 %), sweating (in about 4 %), bradycardia and arterio-ventricular block (in about 1 %) [111, 158].

Oral ambenonium chloride may be used instead of oral pyridostigmin bromide in case of bromide intolerance with gastrointestinal side effects.

### Immunotherapy

The efficacy of immunosuppressive drugs in generalized MG is generally accepted. Moreover, patients with pure ocular myasthenia exhibit a reduced rate of progression to generalized MG when being under immunosuppression [96, 160].

However, only few immunosuppressive drugs have been tested in larger randomized, controlled trials providing unequivocal class I evidence for their use in MG patients. In some randomized, controlled trials positive clinical effects of some immunosuppressive drugs could even not be proven. These studies, however, exhibit methodological weaknesses which will be discussed below.

In contrast, evidence from clinical trials regarding the duration and criteria for cessation of immunotherapy is scarce [82]. Commonly, following several years of stable clinical remission the prolonged tapering of immunosuppressive drug treatment seems possible. Abrupt cessation of immunosuppression especially in a clinically unstable situation may trigger abrupt deterioration of myasthenic symptoms and myasthenic crisis [82, 188]. Indeed, most patients require life-long immunosuppressive treatment which favors opportunistic infections, lymphoma and other severe treatment-associated side effects. The overall aim of symptomatic and immunotherapy should be complete or almost complete clinical remission.

#### Basic immunotherapy

Glucocorticosteroids and azathioprine are first-line drugs for immunosuppression in MG. Other immunosuppressive agents can be used in case of contraindications, intolerability or insufficient clinical disease control during adequately applied first-line therapy. Second-line immunosuppressive drugs are ciclosporine A (1 positive controlled trial [174]), methotrexate (1 positive controlled trial [68]), mycophenolate mofetil (two negative controlled trials although with very short follow-up period [69, 116, 144]) and tacrolimus (1 negative controlled trial although with very short follow-up period [193]).

##### Glucocorticosteroids

In retrospective studies, glucocorticosteroids (GCS) like prednisone, prednisolone and methylprednisolone have shown to improve clinical symptoms within several weeks to months (normally within 4–8 weeks) in about 70–80 % of patients [124, 149, 158]. Due to side effects, long-term oral GCS are usually combined with steroid-sparing immunsuppressive drugs such as azathioprine, cyclosporin A, methotrexate, mycophenolat mofetil or tacrolimus. During the first days after initiation of GCS therapy a transient deterioration of myasthenic symptoms may occur especially in patients with a pronounced affection of bulbar muscle groups [6].

Three different dosing regimens are currently used in clinical practice:

1. Initial dosage of 10–20 mg/day prednisone equivalent and dosage increase of 5 mg/day per week until a stable remission is reached (at about 1 mg/day/kg bodyweight) [153]. Advantage: prevention of a transient deterioration of myasthenic symptoms during the first days of treatment, disadvantage: slow clinical improvement.
2. Start with a dosage of 1–1.5 mg/day/kg bodyweight prednisone equivalent in combination with a steroid-sparing immunosuppressant until induction of stable clinical remission and subsequent dosage reduction of 5 mg/day every 4 weeks with the aim of total cessation of GCS therapy. Advantage: rapid clinical improvement. Disadvantage: Transient deterioration of myasthenic symptoms during the first days of treatment in about 10 % of patients [6, 124].
3. Intravenous methylprednisolone pulse-therapy using 500–2000 mg/day for 3–5 consecutive days followed by an oral taper [2, 101]. This treatment regimen may lead to a transient deterioration of myasthenic symptoms potentially inducing myasthenic crisis due to a direct membrane effect of GCS [37]. Moreover, an acute steroid myopathy may—in some cases—contribute to the overall clinic deterioration in this situation. Hence, this treatment regimen is only used in manifest myasthenic crises and in combination with plasmapheresis, immunoadsorption or intravenous immunoglobuline therapy in an intermediate or intensive care setting.

The number and severity of side effects of GCS increase with the duration and cumulative dosage. Especially patients with other comorbidities, e.g., diabetes mellitus, are at special risk. In case of an estimated therapy duration of longer than 3 months using a dosage of >7.5 mg prednisolone equivalent, patients should be treated with calcium (1000–1500 mg/d) and vitamin D (400–800 IE/d) to prevent osteoporosis. Vitamin D levels should be determined prior to initiation of such therapy and controlled throughout. In post-menopausal women bisphosphonates can be used to prevent GCS-induced osteoporosis. The evidence for the prophylactic effect of bisphosphonates against GCS-induced osteoporosis and bone fractures in men is currently not sufficient for a general recommendation. Moreover, stomach protection using proton-pump inhibitors or other drugs may be warranted.

To reduce side-effects some centers switch from every to alternate day oral GCS treatment during long-term application in the low dose range, the usefulness of which needs to be validated in individual patients as long as systematic data are lacking.

##### Azathioprine

Azathioprine (AZA) is the most frequently used immunosuppressive agent for treatment of MG [13, 66, 105, 112]. Azathioprine is a purine analogue that is metabolized rapidly to the cytotoxic and immunosuppressant derivatives 6-mercaptopurine and thioinosinic acid. The latter inhibits purine synthesis, and thus impairs activation and proliferation and causes apoptosis of T cells and B cells due to their lack of metabolic pathways for nucleotide salvage (‘recycling’). Treatment is initiated using 2–3 mg/day/kg bodyweight and maybe reduced during the treatment course in case of a clinically stable remission to about 2.5 mg/day/kg bodyweight and further to 1 mg/day/kg bodyweight. Treatment effects cannot be expected before several months. However, a steroid-sparing effect of AZA during long-term treatment has been proven [39, 66, 159]. The combination of AZA and prednisolone is more effective, as longer states of remission and less side effects are encountered as compared to respective monotherapies [123]. In 10–20 % of patients the combination of AZA and GCS does not lead to sufficient clinical stabilization or remission requiring GCS at dosages of more than 7.5 mg/day prednisolone equivalent and in the long term requiring other immunosuppressive treatment strategies (therapy resistance). The abrupt cessation of AZA may trigger re-occurence of myasthenic symptoms up to a myasthenic crisis even in patients in complete and stable clinical remission [82, 114].

In about 80 % of patients treated with AZA an increase of the mean corpuscular volume (MCV) of the red blood cells can be observed, which is more pronounced and more frequent in treatment responders compared to non-responders. Due to its mechanism of action, AZA leads to a potentially reversible lymphopenia under steady-state conditions. Absolute lymphocyte counts should be in the range of 600–1200/µl with total leukocyte counts being well above 3500/µl.

AZA is a prodrug that is metabolized by the glutathion-s-transferase into its active metabolite 6-mercaptopurine und 1-methyl-4-nitro-5-thioimidazol. 6-Mercaptopurine in turn is further metabolized by the xanthinoxidase or the thiopurin-S-methyltransferase (TPMT). Inhibitors of the xanthinoxidase such as allopurinol and others inhibit the metabolization of azathioprine. With this comedication azathioprine may only be used in a reduced dosage of 25 % of the standard dosage (i.e., 0.5–0.75 mg/day/kg bodyweight) to prevent myelotoxic side effects. Instead of xanthinoxidase inhibitors, other drugs such as benzbromarone or probenecide may be used for lowering uric acid levels in case AZA is required.

In a small number of patients (<1 %) acute severe side effects like nausea and vomitting, gastrointestinal disturbance with diarrhea and cardiocirculatory depression may occur as idiosyncratic immediate reaction and prevent patients from further treatment with AZA [81]. To exclude such idiosyncratic reactions a single oral test dosage of 50 mg prior to the initiation of long-term treatment with AZA can be recommended to detect such side effects.

In case of a genetically determined low TPMT activity, AZA leads to unexpectedly strong myelosuppression shortly after treatment initiation. Testing for TMTP activity or TPMT genotype can be performed prior to treatment initiation: (a) patients completely lacking TMPT activity (frequency 1:300) or those homozygous for distinct TPMT single nucleotide polymorphisms cannot be treated with AZA. This genotype however is very rare (about 0.5 %) [56]. Whether this genotype is associated with the above mentioned idiosyncratic immediate reaction is currently unclear. (b) In cases of exceedingly high TPMT activity, AZA may be rapidly metabolized without yielding a clinical effect.

An increased risk of malignancies under AZA treatment for less than 10 years is not evident [25, 189]. In patients with MG, rare cases of hematological malignancies and opportunistic infections have been reported [73, 114]. Moreover, an increased incidence of cutaneous hyperkeratosis and skin cancer under azathioprine has been reported, probably due to increased photosensitivity [122]. Regular skin examinations are recommended in patients under long-term therapy with azathioprine. In case of the occurrence of generalized warts, or multiple basalioma, AZA treatment must be reduced or changed to a different drug. A few cases of acute phototoxic reactions have been reported under intense solar irradiation.

##### Ciclosporin A

Ciclosporin A (CSA) has proven its efficacy in patients with MG in one placebo-controlled clinical trial providing class I evidence [174].

Ciclosporin binds to the cytosolic protein cyclophilin in lymphocytes and thereby inhibits the calcium-calmodulin induced activation of calcineurin following antigen-receptor stimulation that is required for antigen-specific lymphocyte activation, differentiation and exertion of effector functions.

Compared to the above mentioned clinical trial (CSA monotherapy using 6 mg/day/kg bodyweight in two single dosages) CSA is currently in clinical use in combination with GCS at a lower dosage of initially 3–4 mg/day/kg bodyweight, followed by a reduction to 2–2.5 mg/day/kg bodyweight in two single dosages. Treatment monitoring should be performed via blood levels (trough level at the end of a dosing interval of 12 h). Compared to AZA, the clinical effect of CSA occurs more rapidly, i.e., mostly within 4–6 weeks. However, CSA has a much broader spectrum of side effects that occur depending on its dosage and include opportunistic infection, myelosuppression, gingival hyperplasia and gastrointestinal disturbance, as well as nephrotoxicity with hyperkaliemia (requiring monitoring of renal function during therapy) and arterial hypertension. Special side effects include tremor, headache, increased propensity of epileptic seizures and rarely reversible posterior leukoencephalopathy syndrome. Moreover, CSA exhibits numerous interactions with other drugs which is especially relevant in elderly, multimorbid patients and requires intense drug monitoring.

##### Methotrexate

Methotrexate (MTX) has been used for several decades now as a treatment for MG. MTX competitively inhibits dihydrofolate reductase (DHFR) with an affinity of about 1000-fold that of dihydrofolate. Dihydrofolate reductase catalyses the conversion of dihydrofolate to the active metabolite tetrahydrofolate involved in the de-novo synthesis of purine and pyrimidine nucleotide synthesis. Methotrexate, therefore, inhibits the synthesis of DNA, RNA, and proteins and thus reduces proliferation among others of lymphocytes. Most prominent side effects of MTX include hepatotoxicity, ulcerative stomatitis, leucopenia, anemia, infections, nausea and vomiting, abdominal pain, acute pneumonitis, and rarely pulmonary fibrosis and kidney failure.

Despite its long-term use in patients with MG, controlled clinical trials were lacking until recently. A recent clinical trial compared MTX (17.5 mg/week) in 24 patients with generalized MG with AZA (2.5 mg/day/kg bodyweight) regarding its steroid-sparing effect and showed an equivalent effect within a treatment period of 2 years [68]. MTX at a dosage of 7.5–25 mg/week in combination with folic acid can thus be regarded as a second-line drug for the treatment of MG. Due to its less frequent interactions with other drugs, MTX may be preferred over CSA especially in older multimorbid patients [76].

##### Mycophenolatmofetil

Mycophenolatmofetil (MMF) non-competitively inhibits the inosin-monophosphate-dehydrogenase (IMPDH) and thus the de-novo synthesis of purine nucleotides which are required especially for cell proliferation in lymphocytes.

The most prominent side effects of MMF include chronic diarrhea, hemolytic anemia and edema. Several cases of progressive multifocal leukoencephalopathy have been reported on the treatment with MMF [183]. Moreover, MMF has proved to be teratogenic. Hence, MMF-treatment should be stopped in case of planned pregnancy at least 4 months before conception. In case of unplanned pregnancy, MMF should be stopped immediately and followed by gynecologic counseling [78].

MG patients showed clinical improvement with steroid-sparing effect under treatment with MMF in several observational cohort studies on a dosage of 1500–2000 mg/day under drug monitoring [21, 64, 69]. However, in two clinical phase III trials, MMF was not superior to prednisone as an initial therapy [116] and did not show any steroid-sparing effect when studied over a period of 9 months [144]. Considering the latency of clinical effects known to occur under MMF therapy, the follow-up period of no longer than 36 weeks in the two phase III studies seems quite short. Moreover, the treatment effect of prednisone was unexpectedly good. Hence, MMF effects seem to be underestimated based on methodological issues in these trials. Consistently, in subsequent uncontrolled cohort studies again a beneficial effect of MMF as monotherapy or in combination with prednisone could be shown after 6 months of treatment [69].

##### Tacrolimus

Tacrolimus (TCM) like CSA is a calcineurin inhibitor effectively inhibiting antigen-specific lymphocyte activation, differentiation and exertion of effector functions in lymphocytes. The efficacy of TCM compared to CSA is 10–100 times stronger. Side effects are the same as those occurring under CSA and exhibit a strong dosage dependency. TCM was developed in Japan and is licensed for the treatment of MG there [118, 172]. Several open clinical studies and smaller case series report beneficial clinical effects of TCM (3–5 mg/day) in patients with therapy-refractory MG [41, 94, 115, 117]. In a multicenter, open cohort study in 79 patients with MG, low-dosage TCM (0.1 mg/day/kg bodyweight) could replace combination therapy consisting of CSA and prednisolon and provide good clinical stabilization including regression of nAChR-Ab titers [130, 131]. A randomized, placebo-controlled clinical trial in 80 patients with MG with minimal clinical disease under an oral prednisolone therapy (10–20 mg/day) studied the steroid-sparing effect of TCM (3 mg/day) over a period of 28 weeks. The oral prednison therapy was reduced stepwise starting after 4 weeks of TCM treatment. However, no significant difference was detected between TCM and placebo regarding the mean oral prednisolone dosage during the last 12 weeks of the follow-up periode. Due to the study population and the short follow up, this study provides little evidence on the long-term efficacy of TCM in patients with MG and insufficient treatment response on the GCS-therapy [193]. Like CSA, TCM is nephro- and neurotoxic and exhibits multiple drug interactions.

#### Escalation therapy

##### Rituximab and other monoclonal antibodies

A number of case reports and case series report on the clinical efficacy of rituximab, a monoclonal anti-CD20 antibody depleting circulating B lymphocytes in patients with severe therapy-refractive MG. However, data from randomized controlled trails is still missing.

A meta-analysis was recently performed of 15 uncontrolled observational studies including a total of 168 patients [125 females and 43 males; 91 MG patients with AChR antibodies, 70 MG patients with MuSK antibodies, and 7 MG patients without AChR or MuSK antibodies (“double seronegative”)] [87]. The median follow-up was 16 months for AChR antibody^+^ patients, 26 months for MuSK antibody^+^ patients and 12 months double seronegative patients. The dosing regimen of rituximab varied between studies: 137 patients received 4 × 375 mg/m^2^ of rituximab, 12 patients received 500 mg on day 1 and 8, and 8 patients received 1000 mg on day 1 and 15. The remaining 11 patients underwent different treatment regimens. The overall response rate was reported to be 83.9 %. In particular the response rate was higher in the MuSK antibody^+^ patients (88.8 %) compared to AChR antibody^+^ patients (80.4 %) and double seronegative patients (85.6 %). However these differences between different MG patients groups were not statistically significant. Rituximab proved to be efficacious in all different therapeutic regimens. It has been hypothesized that IgG4 antibodies against MuSK are produced almost exclusively by CD20^+^ short-lived plasma cells in contrast to IgG1 and 3 antibodies against AChR which seem to be synthesized by CD20^−^ long-lived plasma cells. This might explain the by trend higher efficacy of rituximab in MuSK antibody^+^ as compared to AChR antibody^+^ patients [32, 84]. In MuSK antibody^+^ patients clinically responsive to rituximab antibody titers are typically lowered whereas some dissociation between antibody titers and clinical response to rituximab was observed in AChR antibody^+^ patients [19].

Moreover, an inverse correlation trend between disease duration and response rate to rituximab was observed which also did not reach statistical significance. This trend was also attributed to the pool of long-lived plasma cells increasing with disease duration and thus leading to a diminishing therapeutic effect of rituximab. Adverse effects were reported in 7/168 patients (4.2 %; infections in 4 patients (herpes zoster 1, giardiasis 1, bronchitis 1, pneumonia 1), prolonged B cell depletion in 2 patients, and heart failure in 1 patient). No case of treatment-associated progressive multifocal leukoencephalopathy was reported. Hence, data from this meta-analysis of uncontrolled observational studies support the use of rituximab in patients with refractory MG. Nevertheless, multicentre randomized-controlled trials are needed to definitely establish the efficacy and safety of rituximab in MG.

Eculizumab is a humanized monoclonal antibody, blocking the formation of terminal complement complex by specifically preventing the enzymatic cleavage of complement 5 (C5). A recent, randomized, double-blind, placebo-controlled, crossover phase II trial studied the clinical efficacy of eculizumab in 14 patients with severe, refractory generalized MG [83]. Patients received 4 weekly intravenous infusions of eculizumab 600 mg or placebo (induction phase) followed by seven additional two weekly intravenous infusions of eculizumab 900 mg or placebo (maintenance phase). Six of seven patients treated with eculizumab for 16 weeks (86 %) achieved the primary endpoint of a 3-point reduction in the quantitative myasthenia gravis (QMG) score. Moreover, the overall change in mean QMG total score was significantly different between eculizumab and placebo, and the overall change in mean QMG total score from baseline was found to be significantly different between eculizumab and placebo. Eculizumab was well tolerated [83]. Hence, this trial supports a role also for eculizumab in patients with severe, refractory generalized MG. A Phase III trial of eculizumab in refractory MG is currently ongoing [126].

Clinical experience with other therapeutic monoclonal antibodies now in broader use in multiple sclerosis such as anti-CD52/alemtuzumab [22, 24] and anti-IL2R/daclizumab [54, 58] are sparce in patients with MG and should be considered in individual rare cases only.

##### Cyclophosphamide

Cyclophosphamide (CPP) is a nitrogen mustard alkylating agent adding an alkylgroup to DNA. This interferes with DNA replication by forming intrastrand and interstrand DNA crosslinks. CPP can be applied in cases of severe MG following the failure of standard therapy. Moreover, CPP can be applied in patients requiring repetitive immunoadsorption or plasmapheresis based on several smaller controlled case series [35, 100]. In severe and otherwise treatment resistant life-threatening MG, CPP can be used as ultima ratio as positive clinical evidence exist for several treatment regimens:

1. CPP pulse therapy: 500 mg/m^2^ body surface area every 4 weeks until remission under co-medication with mesna, based on a prospective, randomized, double-blinded study [31].
2. Immuno- or myeloablative CPP therapy: 50 mg/day/kg bodyweight on 4 consecutive days followed by GCSF based on several smaller case studies [36, 57, 100] or followed by auto- or allogeneic stem cell transplantation [168].

The cumulative dosage and duration of treatment should be documented due to the increasing risk of disorders of fertility in both sexes and the risk of malignoma (about 1 %, depending on the cumulative dosage and duration of therapy). In single cases, a cumulative dosage of 50–70 g can be reached. As these values are reached much faster upon oral application compared to intravenous application of CPP, the later should be the preferred route of administration. Complications of CPP therapy may among others include malignoma, lung fibrosis, cardiomyopathy and dermatofibroma.

For an overview on treatment options for MG please refer to Table 2.

**Table 2:** Treatment options for MG

Substances	Dose	Side effects	Contraindications and special remarks
Cholinesterase inhibitors			
Pyridostigmin bromide (approved)	Single dose orally: 30–60 mg max. 360 mg/day	Stimulation of muscarinic AChR (smooth muscles, gland secretion): stomach crampi, nausea, vomiting, anorexia, diarrhea, urinary urgency, salivation/lacrivation, sweating, bronchial secretion, accommodation errors, miosis, bradycardia (rarely AV block), hypotonia stimulation of nicotinic AChR (skeletal muscles): muscle fasciculation, spasms, muscle weakness (depolarization block)	Absolute contraindications: asthma bronchiale, prostata hypertrophy, decompensated heart insufficiency, acute myocardial infarction, thyreotoxicosis relative contraindications: pregnancy, breast-feeding
Ambenonium chloride (off label)	Single dose orally 5–10 mg max. 40 mg/day	Fewer gastrointestinal side effects than pyridostigmin	Analog to pyridostigmin
Immunosuppressants			
Glucocorticosteroids: prednisone prednisolone methylprednisolone (approved)	Oral therapy: 0.5–1.5 mg/kg Intravenous pulse therapy: 500–1000 mg/day for 1–3 days cave: initial transient deterioration especially of bulbar myasthenic symptoms—ICU monitoring and therapy necessary	Gain of weight, cushingoid phenotype, acne, diabetes, susceptibility to infections and thrombosis, hypertonia, hypokaliema, edema, psychosis, osteoporosis with the risk of fractures, aseptic bone necrosis, cataract, glaucoma, psychological disorders (euphoria/depression), insomnia, steroid myopathy, gastric and duodenal ulcera	Severe infections, malignant diseases, severely reduced immune defense, pregnancy, breast-feeding severe infections, manifest gastric and duodenal ulcera, severe osteoporosis, psychiatric disorders, uncontrolled hypertonia, uncontrolled diabetes
Azathioprine (approved)	Induction dose: 2–3 mg/day/kg Maintenance dose: 1.5–2 mg/day/kg	Susceptibility to infections, bone marrow depression (leukopenia, thrombopenia, rarely anemia), nausea, vomiting, diarrhea, fever, allergic reaction, hepatotoxicity, arthralgia, myalgia, alveolitis, pancreatitis, skin exanthema	Pregnancy: azathioprine may be prescribed in case of an appropriate indication. If a female patient is stable on azathioprine therapy should not be stopped Breast-feeding: azathioprine therapy does not exclude breast feeding No vaccinations with live vaccines! Success of vaccinations in general is uncertain Simultaneous admission of allopurinol or other xantinoxidase inhibitors leads to myelotoxicity and agranulocytosis—dose reduction to 25 % of azathioprine or switch from allopurinol to probenezid or benzbromaron severe bone marrow and liver and kidney damage
Ciclosporin A (off-label)	2 (−5) mg/day/kg in two single doses	Hypertonia, nephrotoxicity (nephropathy, hyperkalemia), CNS-toxicity (tremor, paresthesia, seizures), posterior reversible encephalopathy syndrome, hepatotoxicity, hirsutism, gingiva hyperplasia, secondary malignancies, infections	Kidney failure, severe infections, malignant diseases, severely reduced immune defense, pregnancy, breast-feeding
Methotrexate (off-label)	7.5–15 mg once per week max. 25 mg once per week in combination with folic acid (5 mg) 24 h after application	Hepatotoxicity, bow marrow depression, gastrointestinal symptoms, stomatitis, ulcera, exanthema, loss of hair, hyperuricemia, kidney failure, cystitis, lung fibrosis, cutaneous vasculitis, photosensitivity, psychiatric disorder, osteoporosis	Liver or kidney failure, severe infections, malignant diseases, severely reduced immune defense, pregnancy, breast-feeding, bone marrow depression, florid gastrointestinal ulcera
Mycophenolat mofetil (off-label)	0.5–3 g/day in two single doses (mostly 2 × 1 g/day)	Gastrointestinal symptoms (nausea, vomiting, diarrhea, ulcera, gastrointestinal bleeding), bone marrow depression (leukopenia, anemia, thrombocytopenia), infections, risk for lymphoma under long-term therapy, progressive multifocal leukoencephalopathy (PML)	Severe infections, malignant diseases, severely reduced immune defense, pregnancy, breast-feeding
Tacrolimus (off-label)	0.1–0.2 mg/day/kg in two single doses	Hypertonia, nephrotoxicity (nephropathy, hyperkalemia), ZNS-toxicity (tremor, paresthesia, seizures), posterior reversible encephalopathy syndrome, hepatotoxicity, hirsutism, gingivahyperplasia, secondary malignancies, infections	Kidney failure, severe infections, malignant diseases, severely reduced immune defense, pregnancy, breast-feeding
Rituximab (off-label)	1000 mg i.v. at day 1 and 15 every 6–9 months	Infusion reaction within 24 days after, infections (upper and lower respiratory tract, urinary infections), Lyell Syndrome (toxic epidermal necrolysis), Stevens-Johnson Syndrome, progressive multifocal leukoencephalopathy (PML)	Severe infections, malignant diseases, severely reduced immune defense, pregnancy, breast-feeding
Cyclophosphamide (off-label)	Intravenous pulse therapy: 500–750 mg/m^2^ i.v. every 4–8 weeks under urothelial protection with mesna Oral therapy is not recommended due to side effects and rapidly high cumulative doses (maximal empiric cumulative dose 50–70 g)	Bone marrow depression, gastrointestinal symptoms, cystitis (adequate hydration!), loss of hair, liver and kidney damage, dermatitis, stomatitis, hyperuricemia elevated incidence of secondary malignancies	Kidney failure, severe infections, malignant diseases, severely reduced immune defense, pregnancy, breast-feeding

### Intervention therapy

The following therapeutic measures are applied for the prevention and therapy of myasthenic crisis and in special situations such as instable MG during pregnancy and distinct single cases of therapy resistant myasthenia with severely disabling symptoms.

#### Intravenous immunoglobulins (IVIG)

Intravenous immunoglobulins (IVIG) consist of pooled polyclonal immunoglobulins derived from several thousand healthy donors. The precise mechanism by which IVIGs suppress autoimmune inflammation has not been definitively established but is likely to involve a plethora of molecular effects via their Fab- or Fc-fragments [52]. Adverse effects of IVIG include headache, hypertension, allergic/anaphylactic reactions (especially in IgA-deficient patients), dermatitis, infection (HIV or viral hepatitis), pulmonary edema from fluid overload, due to the high colloid oncotic pressure of IVIGs, venous thrombosis, aseptic meningitis, and hemolysis.

IVIG should be applied at a dosage of 0.4 g/day/kg bodyweight on 5 consecutive days [86], alternatively 1 g/day/kg bodyweight on 2 consecutive days [7, 50, 198]. IVIG have been shown to be similarly effective compared to plasmapheresis and immunoadsorption in shortening the time of mechanical ventilation during myasthenic crisis [46, 49].

Moreover, IVIG may be used for clinical stabilization previous to operations (including thymectomy) or before start of an intravenous high-dosage GCS pulse therapy in case of severe MG. Clinical response rate in several open clinical studies was in the range of 80 %.

For the use of IVIG in situations other than myasthenic crisis as induction therapy or as maintenance therapy—either alone or as add-on therapy to immunosuppressive drugs—no data is available from randomized controlled trials. IVIG, however, may be used for this purpose (initially 5 × 0.4 g/day/kg bodyweight and subsequently 1 × 0.4 g/day/kg bodyweight every 4–8 weeks) over a longer period of time [38, 72, 161]. Single patients with therapy resistant myasthenic symptoms seem to profit from long-term therapy with IVIG [111, 112]. IVIG may also be used in MG patients with contraindications against other immunosuppressive agents (especially GCS).

#### Plasmapheresis and immunoadsorption

Via therapeutic plasma exchange (PE) or plasmapheresis, plasma is separated from corpuscular blood constituents and replaced with a substitution fluid. PE is thus a non-specific treatment modality with elimination of the entire plasma. The therapeutic effect is based on the removal of circulating, pathogenic immune factors, including autoantibodies. In contrast, immunoadsorption (IA) is a more selective technique for removing IgG antibodies by binding to a specific matrix (e.g., protein A or tryptophan) [95, 150, 180].

Plasmapheresis is successfully used as a treatment of myasthenic crisis [30, 127, 142]. Moreover, plasmapheresis can be used in situations of therapy resistance for the stabilization of patients before operations (including thymectomy) or prior to the initiation of high-dosage GCS pulse therapy in cases of severe MG. Typically, 6–8 treatments (each with treatment of 1–1.5 times the plasma volume every other day) are performed until clinical remission is reached. Without accompanying immunosuppression the clinical effect lasts for several weeks only due to the (re-)synthesis of pathogenic autoantibodies [70, 119]. Following every single treatment, a substitution of human albumin is required, and in case of secondary immunoglobuline deficiency (IgG < 150 mg/dl) substitution with polyvalent IgG can be performed. The depletion also of coagulation factors limits the treatment frequency and needs to be considered in situations of treatment with other anticoagulant drugs [48]. In myasthenic crisis, plasmaphereses and IVIG seem to be equally effective and can be used equivalently [26, 47, 158]. A randomized controlled study did not find a significant difference between both therapeutic strategies [46]. Moreover, a controlled cross-over study and a retrospective cohort study did also show no significant difference between these treatments [38, 72, 133, 161]. A recent comparative study in 84 patients with moderate to severe MG (QMGS >10.5) and clinical exacerbation also showed a similar efficacy of IVIG and plasmapheresis regarding the primary endpoint of reduction of the QMGS (69 % for IVIG and 65 % for plasmapheresis) as well as secondary clinical and electrophysiological endpoints for a follow-up period of 60 days [10].

Immunoadsorption is nowadays frequently used instead of plasmapheresis both for the treatment of myasthenic crisis as well as for the maintenance therapy of patients with insufficient disease control under or contraindications for standard immunosuppressive treatment [191, 194]. Immunoadsorption has been shown to be equally effective in the treatment of MG compared to plasmapheresis [93]. Advantages of immunoadsorption include the lack of need for substitution of plasma proteins and coagulation factors, providing the option for the rapid treatment of much higher plasma volumes (2–2.5 times the plasma volume every day) as compared to plasmapheresis. Moreover, complications and side effects of immunoadsorption seem to be significantly reduced compared to plasmapheresis [93].

## Thymectomy

Thymectomy in MG patients should always be performed under otherwise stable clinical conditions, i.e., following an efficient pre-treatment using GCS and other immunosuppressive measures and/or plasmapheresis/immunoadsorption or IVIG which reduces the perioperative mortality to <1 % [61]. The clinical effect of thymectomy usually appears retrospectively after several years in an individual patient [61].

### Thymomectomy for thymomatous MG

In case of a thymoma, indication for thymomectomy exists independent of the presence or clinical severity of accompanying MG or other autoimmune conditions.

### Thymectomy for non-thymomatous AChR antibody associated MG

Given the role of the thymus in the immunopathogenesis of AchR antibody-associated MG, thymectomy has been considered a treatment option as early as 1942 [67]. However, randomized controlled trials on the efficacy of thymectomy per se and in comparison to standard immunosuppressive treatment in MG are currently not available [18]. The results of an ongoing multi-center, single-blind, randomized phase III trial comparing extended transsternal thymectomy to no thymectomy in non-thymomatous AChR antibody associated MG patients receiving prednisone (MGTX trial [12, 190]) are expected for 2016.

Until the results of the MGTX trial are available, thymectomy should be considered in the following clinical situations: (a) Thymectomy may be applied in patients with non-thymomatous generalized MG for which evidence exists according to a meta-analysis [61]. (b) Patients with non-thymomatous ocular myasthenia may undergo thymectomy based on a single case decision due to a lack of sufficient evidence from clinical trials [11]. It is supposed that thymectomy in ocular myasthenia without thymoma might prevent generalization of myasthenic symptoms during the course of the disease [89].

Patients at the age of 15–50 years with generalized AchR antibody-associated MG without thymoma seem to predominantly benefit from thymectomy performed within 1–2 years after disease onset [61, 152]. However, these age limits are arbitrary and some experts do not consider them strict [80]. Children and adolescents at the age of 5–14 years with AchR antibody-associated MG without thymoma are preferred to undergo thymectomy only after insufficient response to symptomatic and immunosuppressive treatment [16, 45] because of the role of the thymus in the development of the immune system [177]. However, some studies indicate that thymectomy at 1.5 years of age apparently does not impair immunological function [4, 136, 177].

### Thymectomy for non-thymomatous MuSK antibody associated MG

In contrast to AchR antibody-associated MG, thymic pathology is relatively rare [63, 97, 99, 197] in patients with MuSK antibody-associated MG. Accordingly, one study [42] could not prove any effect of thymectomy in 15 MuSK antibody-positive patients, whilst MuSK antibodies predicted a poor outcome of thymectomy in another study [129]. Hence, available evidence suggests that in general thymectomy should not be recommended in MuSK antibody-associated MG. However, as few MuSK antibody-positive patients have been reported to apparently improve following thymectomy it may be considered in single cases with otherwise poor disease control [42, 143].

### Thymectomy for non-thymomatous seronegative MG

A retrospective cohort study of thymectomy reported similar post-operative results in AChR antibody-negative and AChR antibody-positive MG patients with a follow-up of at least 3 years [62]. Remission or improvement after thymectomy occurred in 57 % of AChR antibody-negative patients and in 51 % of AChR antibody-positive patients. Hence, thymectomy is recommended for patients with generalized MG without detectable AChR and MuSK antibodies similar to those patients with AChR antibodies.

### Technique of thymectomy

Standard procedure for thymectomy used to be the extended transsternal thymectomy with excision of the whole thymus and retrosternal adipose tissue aiming at a maximal thymectomy [88]. However, minimal invasive interventions are increasingly applied [5, 53, 121, 140, 141, 192]. While these studies cannot be compared to each other due to pronounced confounding factors, the reported effects on therapeutic and clinic surrogates seem to be equivalent [113]. Hence minimally invasive thymectomy represents an option alternative to the gold standard of extended transternal thymectomy and is used by an increasing number of centers.

A suggested treatment regimen is depicted in Table 3.

**Table 3:**
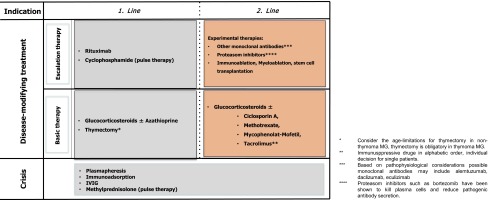
Treatment strategies for MG

## Conclusion

Myasthenia gravis (MG) is an autoimmune antibody-mediated disorder of neuromuscular synaptic transmission. The clinical hallmark of MG consists of fluctuating fatigability and weakness affecting ocular, bulbar and (proximal) limb skeletal muscle groups. MG may either occur as an autoimmune disease with distinct immunogenetic characteristics or as a paraneoplastic syndrome associated with tumors of the thymus. Impairment of central thymic and peripheral self-tolerance mechanisms in both cases is thought to favor an autoimmune CD4^+^ T cell-mediated B cell activation and synthesis of pathogenic high-affinity autoantibodies of either the IgG1 and 3 or IgG4 subclasses. These autoantibodies bind to the nicotinic acetylcholine receptor (AchR) itself, or muscle-specific tyrosine-kinase (MuSK), lipoprotein receptor-related protein 4 (LRP4) and agrin involved in clustering of AchRs within the postsynaptic membrane and structural maintenance of the neuromuscular synapse.

Treatment strategies for MG can be divided into: (a) symptomatic treatments with AChEIs facilitating neuromuscular transmission, (b) antibody-depleting treatments (IVIG, plasmapheresis, or immunoadsorption) for acute therapeutic intervention, and (c) immunotherapeutic treatment strategies (GCS plus azathioprine, ciclosporin A, methotreate, mycophenolat mofetil, or tacrolimus for basic therapy, cyclophosphamide or rituximab for escalation therapy) for maintenance therapy. Experimental therapies such as other monoclonal antibodies, proteasom inhibitors, immune—or myeloablation, and stem cell transplantation are used in selected rare cases only. Given the role of the thymus in the immunopathogenesis of EOMG and TAMG, thymectomy should be performed following sufficient clinical stabilization.

## References

[1] Albuquerque EX, Pereira EF, Alkondon M, Rogers SW (2009). Mammalian nicotinic acetylcholine receptors: from structure to function. Physiol Rev.

[2] Arsura E, Brunner NG, Namba T, Grob D (1985). High-dose intravenous methylprednisolone in myasthenia gravis. Arch Neurol.

[3] Aschenbrenner K, D’Cruz LM, Vollmann EH, Hinterberger M, Emmerich J, Swee LK, Rolink A, Klein L (2007). Selection of Foxp3+ regulatory T cells specific for self antigen expressed and presented by Aire + medullary thymic epithelial cells. Nat Immunol.

[4] Ashraf VV, Taly AB, Veerendrakumar M, Rao S (2006). Myasthenia gravis in children: a longitudinal study. Acta Neurol Scand.

[5] Bachmann K, Burkhardt D, Schreiter I, Kaifi J, Busch C, Thayssen G, Izbicki JR, Strate T (2008). Long-term outcome and quality of life after open and thoracoscopic thymectomy for myasthenia gravis: analysis of 131 patients. Surg Endosc.

[6] Bae JS, Go SM, Kim BJ (2006). Clinical predictors of steroid-induced exacerbation in myasthenia gravis. J Clin Neurosci.

[7] Bain PG, Motomura M, Newsom-Davis J, Misbah SA, Chapel HM, Lee ML, Vincent A, Lang B (1996). Effects of intravenous immunoglobulin on muscle weakness and calcium-channel autoantibodies in the Lambert-Eaton myasthenic syndrome. Neurology.

[8] Balandina A, Lecart S, Dartevelle P, Saoudi A, Berrih-Aknin S (2005). Functional defect of regulatory CD4(+)CD25+ T cells in the thymus of patients with autoimmune myasthenia gravis. Blood.

[9] Barik A, Lu Y, Sathyamurthy A, Bowman A, Shen C, Li L, Xiong WC, Mei L (2014). LRP4 is critical for neuromuscular junction maintenance. J Neurosci.

[10] Barth D, Nabavi Nouri M, Ng E, Nwe P, Bril V (2011). Comparison of IVIg and PLEX in patients with myasthenia gravis. Neurology.

[11] Benatar M, Kaminski H (2012). Medical and surgical treatment for ocular myasthenia. Cochrane Database Syst Rev.

[12] Birmingham UoAa (2006) Thymectomy Trial in Non-Thymomatous Myasthenia Gravis Patients Receiving Prednisone Therapy. ClinicalTrialsgov NCT00294658

[13] Bromberg MB, Wald JJ, Forshew DA, Feldman EL, Albers JW (1997). Randomized trial of azathioprine or prednisone for initial immunosuppressive treatment of myasthenia gravis. J Neurol Sci.

[14] Buckley C, Douek D, Newsom-Davis J, Vincent A, Willcox N (2001). Mature, long-lived CD4+ and CD8+ T cells are generated by the thymoma in myasthenia gravis. Ann Neurol.

[15] Carr AS, Cardwell CR, McCarron PO, McConville J (2010). A systematic review of population based epidemiological studies in Myasthenia Gravis. BMC Neurol.

[16] Castro D, Derisavifard S, Anderson M, Greene M, Iannaccone S (2013). Juvenile myasthenia gravis: a twenty-year experience. J Clin Neuromuscul Dis.

[17] Cavalcante P, Bernasconi P, Mantegazza R (2012). Autoimmune mechanisms in myasthenia gravis. Curr Opin Neurol.

[18] Cea G, Benatar M, Verdugo RJ, Salinas RA (2013). Thymectomy for non-thymomatous myasthenia gravis. Cochrane Database Syst Rev.

[19] Chan A, Lee DH, Linker R, Mohr A, Toyka KV, Gold R (2007). Rescue therapy with anti-CD20 treatment in neuroimmunologic breakthrough disease. J Neurol.

[20] Chuang WY, Strobel P, Bohlender-Willke AL, Rieckmann P, Nix W, Schalke B, Gold R, Opitz A, Klinker E, Inoue M, Muller-Hermelink HK, Saruhan-Direskeneli G, Bugert P, Willcox N, Marx A (2014). Late-onset myasthenia gravis—CTLA4(low) genotype association and low-for-age thymic output of naive T cells. J Autoimmun.

[21] Ciafaloni E, Massey JM, Tucker-Lipscomb B, Sanders DB (2001). Mycophenolate mofetil for myasthenia gravis: an open-label pilot study. Neurology.

[22] Cohen JA, Coles AJ, Arnold DL, Confavreux C, Fox EJ, Hartung HP, Havrdova E, Selmaj KW, Weiner HL, Fisher E, Brinar VV, Giovannoni G, Stojanovic M, Ertik BI, Lake SL, Margolin DH, Panzara MA, Compston DA (2012). Alemtuzumab versus interferon beta 1a as first-line treatment for patients with relapsing-remitting multiple sclerosis: a randomised controlled phase 3 trial. Lancet.

[23] Cole RN, Reddel SW, Gervasio OL, Phillips WD (2008). Anti-MuSK patient antibodies disrupt the mouse neuromuscular junction. Ann Neurol.

[24] Coles AJ, Twyman CL, Arnold DL, Cohen JA, Confavreux C, Fox EJ, Hartung HP, Havrdova E, Selmaj KW, Weiner HL, Miller T, Fisher E, Sandbrink R, Lake SL, Margolin DH, Oyuela P, Panzara MA, Compston DA (2012). Alemtuzumab for patients with relapsing multiple sclerosis after disease-modifying therapy: a randomised controlled phase 3 trial. Lancet.

[25] Confavreux C, Saddier P, Grimaud J, Moreau T, Adeleine P, Aimard G (1996). Risk of cancer from azathioprine therapy in multiple sclerosis: a case-control study. Neurology.

[26] Cortese I, Chaudhry V, So YT, Cantor F, Cornblath DR, Rae-Grant A (2011). Evidence-based guideline update: plasmapheresis in neurologic disorders: report of the Therapeutics and Technology Assessment Subcommittee of the American Academy of Neurology. Neurology.

[27] Costa J, Evangelista T, Conceicao I, de Carvalho M (2004). Repetitive nerve stimulation in myasthenia gravis–relative sensitivity of different muscles. Clin Neurophysiol.

[28] Curnow J, Corlett L, Willcox N, Vincent A (2001). Presentation by myoblasts of an epitope from endogenous acetylcholine receptor indicates a potential role in the spreading of the immune response. J Neuroimmunol.

[29] Dardenne M, Savino W, Bach JF (1987). Thymomatous epithelial cells and skeletal muscle share a common epitope defined by a monoclonal antibody. Am J Pathol.

[30] Dau PC, Lindstrom JM, Cassel CK, Denys EH, Shev EE, Spitler LE (1977). Plasmapheresis and immunosuppressive drug therapy in myasthenia gravis. N Engl J Med.

[31] De Feo LG, Schottlender J, Martelli NA, Molfino NA (2002). Use of intravenous pulsed cyclophosphamide in severe, generalized myasthenia gravis. Muscle Nerve.

[32] Diaz-Manera J, Martinez-Hernandez E, Querol L, Klooster R, Rojas-Garcia R, Suarez-Calvet X, Munoz-Blanco JL, Mazia C, Straasheijm KR, Gallardo E, Juarez C, Verschuuren JJ, Illa I (2012). Long-lasting treatment effect of rituximab in MuSK myasthenia. Neurology.

[33] Diaz-Manera J, Rojas-Garcia R, Gallardo E, Juarez C, Martinez-Domeno A, Martinez-Ramirez S, Dalmau J, Blesa R, Illa I (2007). Antibodies to AChR, MuSK and VGKC in a patient with myasthenia gravis and Morvan’s syndrome. Nat Clin Pract Neurol.

[34] Douek DC, McFarland RD, Keiser PH, Gage EA, Massey JM, Haynes BF, Polis MA, Haase AT, Feinberg MB, Sullivan JL, Jamieson BD, Zack JA, Picker LJ, Koup RA (1998). Changes in thymic function with age and during the treatment of HIV infection. Nature.

[35] Drachman DB, Adams RN, Hu R, Jones RJ, Brodsky RA (2008). Rebooting the immune system with high-dose cyclophosphamide for treatment of refractory myasthenia gravis. Ann NY Acad Sci.

[36] Drachman DB, Jones RJ, Brodsky RA (2003). Treatment of refractory myasthenia: “rebooting” with high-dose cyclophosphamide. Ann Neurol.

[37] Dudel J, Birnberger KL, Toyka KV, Schlegel C, Besinger U (1979). Effects of myasthenic immunoglobulins and of prednisolone on spontaneous miniature end-plate potentials in mouse diaphragms. Exp Neurol.

[38] Eienbroker C, Seitz F, Spengler A, Kurz H, Seipelt M, Sommer N, Oertel WH, Timmesfeld N, Tackenberg B (2014) IVIg maintenance treatment in myasthenia gravis—a RCT sample size simulation. Muscle Nerve 10.1002/mus.24259 24710856

[39] Evoli A, Batocchi AP, Minisci C, Di Schino C, Tonali P (2000). Clinical characteristics and prognosis of myasthenia gravis in older people. J Am Geriatr Soc.

[40] Evoli A, Bianchi MR, Riso R, Minicuci GM, Batocchi AP, Servidei S, Scuderi F, Bartoccioni E (2008). Response to therapy in myasthenia gravis with anti-MuSK antibodies. Ann NY Acad Sci.

[41] Evoli A, Di Schino C, Marsili F, Punzi C (2002). Successful treatment of myasthenia gravis with tacrolimus. Muscle Nerve.

[42] Evoli A, Tonali PA, Padua L, Monaco ML, Scuderi F, Batocchi AP, Marino M, Bartoccioni E (2003). Clinical correlates with anti-MuSK antibodies in generalized seronegative myasthenia gravis. Brain.

[43] Finn R, Coates PM (1977). Plasma exchange in myasthenia gravis. Lancet.

[44] Fuhr P, Gold R, Hohlfeld R, Melms A, Melzer N, Tackenberg B, Schalke B, Schneider-Gold C, Wiendl H, Zimprich F (2014) Diagnostik und Therapie der Myasthenia gravis und des Lambert-Eaton-Syndroms. Leitlinien der Deutschen Gesellschaft für Neurologie

[45] Gadient P, Bolton J, Puri V (2009). Juvenile myasthenia gravis: three case reports and a literature review. J Child Neurol.

[46] Gajdos P, Chevret S, Clair B, Tranchant C, Chastang C (1997). Clinical trial of plasma exchange and high-dose intravenous immunoglobulin in myasthenia gravis. Myasthenia Gravis Clinical Study Group. Ann Neurol.

[47] Gajdos P, Chevret S, Toyka K (2008) Intravenous immunoglobulin for myasthenia gravis. Cochrane Database Syst Rev CD002277 10.1002/14651858.CD002277.pub4 PMC7133495 23235588

[48] Gajdos P, Chevret S, Toyka K (2002) Plasma exchange for myasthenia gravis. Cochrane Database Syst Rev CD002275 10.1002/14651858.CD002275 PMC8985203 12519572

[49] Gajdos P, Chevret S, Toyka KV (2012). Intravenous immunoglobulin for myasthenia gravis. Cochrane Database Syst Rev.

[50] Gajdos P, Tranchant C, Clair B, Bolgert F, Eymard B, Stojkovic T, Attarian S, Chevret S, Myasthenia Gravis Clinical Study G (2005). Treatment of myasthenia gravis exacerbation with intravenous immunoglobulin: a randomized double-blind clinical trial. Arch Neurol.

[51] Gasperi C, Melms A, Schoser B, Zhang Y, Meltoranta J, Risson V, Schaeffer L, Schalke B, Kroger S (2014). Anti-agrin autoantibodies in myasthenia gravis. Neurology.

[52] Gelfand EW (2012). Intravenous immune globulin in autoimmune and inflammatory diseases. N Engl J Med.

[53] Gellert K, Bottger J, Martin T, Werner J, Mangler C, Martin H (2005). Thoracoscopic thymectomy in the treatment concept for myasthenia gravis. Surg Technol Int.

[54] Giovannoni G, Gold R, Selmaj K, Havrdova E, Montalban X, Radue EW, Stefoski D, McNeill M, Amaravadi L, Sweetser M, Elkins J, O’Neill G (2014). Daclizumab high-yield process in relapsing-remitting multiple sclerosis (SELECTION): a multicentre, randomised, double-blind extension trial. Lancet Neurol.

[55] Giraud M, Taubert R, Vandiedonck C, Ke X, Levi-Strauss M, Pagani F, Baralle FE, Eymard B, Tranchant C, Gajdos P, Vincent A, Willcox N, Beeson D, Kyewski B, Garchon HJ (2007). An IRF8-binding promoter variant and AIRE control CHRNA1 promiscuous expression in thymus. Nature.

[56] Gisbert JP, Gomollon F, Cara C, Luna M, Gonzalez-Lama Y, Pajares JM, Mate J, Guijarro LG (2007). Thiopurine methyltransferase activity in Spain: a study of 14,545 patients. Dig Dis Sci.

[57] Gladstone DE, Brannagan TH, Schwartzman RJ, Prestrud AA, Brodsky I (2004). High dose cyclophosphamide for severe refractory myasthenia gravis. J Neurol Neurosurg Psychiatry.

[58] Gold R, Giovannoni G, Selmaj K, Havrdova E, Montalban X, Radue EW, Stefoski D, Robinson R, Riester K, Rana J, Elkins J, O’Neill G (2013). Daclizumab high-yield process in relapsing-remitting multiple sclerosis (SELECT): a randomised, double-blind, placebo-controlled trial. Lancet.

[59] Goldstein G, Whittingham S (1966). Experimental autoimmune thymitis. An animal model of human myasthenia gravis. Lancet.

[60] Gregersen PK, Kosoy R, Lee AT, Lamb J, Sussman J, McKee D, Simpfendorfer KR, Pirskanen-Matell R, Piehl F, Pan-Hammarstrom Q, Verschuuren JJ, Titulaer MJ, Niks EH, Marx A, Strobel P, Tackenberg B, Putz M, Maniaol A, Elsais A, Tallaksen C, Harbo HF, Lie BA, Raychaudhuri S, de Bakker PI, Melms A, Garchon HJ, Willcox N, Hammarstrom L, Seldin MF (2012). Risk for myasthenia gravis maps to a (151) Pro– > Ala change in TNIP1 and to human leukocyte antigen-B*08. Ann Neurol.

[61] Gronseth GS, Barohn RJ (2000). Practice parameter: thymectomy for autoimmune myasthenia gravis (an evidence-based review): report of the Quality Standards Subcommittee of the American Academy of Neurology. Neurology.

[62] Guillermo GR, Tellez-Zenteno JF, Weder-Cisneros N, Mimenza A, Estanol B, Remes-Troche JM, Cantu-Brito C (2004). Response of thymectomy: clinical and pathological characteristics among seronegative and seropositive myasthenia gravis patients. Acta Neurol Scand.

[63] Guptill JT, Sanders DB, Evoli A (2011). Anti-MuSK antibody myasthenia gravis: clinical findings and response to treatment in two large cohorts. Muscle Nerve.

[64] Hanisch F, Wendt M, Zierz S (2009). Mycophenolate mofetil as second line immunosuppressant in Myasthenia gravis–a long-term prospective open-label study. Eur J Med Res.

[65] Hapnes L, Willcox N, Oftedal BE, Owe JF, Gilhus NE, Meager A, Husebye ES, Wolff AS (2012). Radioligand-binding assay reveals distinct autoantibody preferences for type I interferons in APS I and myasthenia gravis subgroups. J Clin Immunol.

[66] Hart IK, Sathasivam S, Sharshar T (2007) Immunosuppressive agents for myasthenia gravis. Cochrane Database Syst Rev:CD005224 10.1002/14651858.CD005224.pub2 PMC13178726 17943844

[67] Harvey AM, Lilienthal JL, Talbot SA (1942). Observations on the nature of myasthenia gravis. The effect of thymectomy on neuro-muscular transmission. J Clin Invest.

[68] Heckmann JM, Rawoot A, Bateman K, Renison R, Badri M (2011). A single-blinded trial of methotrexate versus azathioprine as steroid-sparing agents in generalized myasthenia gravis. BMC Neurol.

[69] Hehir MK, Burns TM, Alpers J, Conaway MR, Sawa M, Sanders DB (2010). Mycophenolate mofetil in achr-antibody-positive myasthenia gravis: outcomes in 102 patients. Muscle Nerve.

[70] Heininger K, Hendricks M, Toyka KV (1985). Myasthenia-gravis—a new semiselective procedure to remove acetylcholine-receptor-autoantibodies from plasma. Plasma Ther Trans Technol.

[71] Hemminki K, Li X, Sundquist K (2006). Familial risks for diseases of myoneural junction and muscle in siblings based on hospitalizations and deaths in sweden. Twin Res Hum Genet.

[72] Henze T, Janzen RWC, Schumm F, Melms A, Sieb JP, Kohler W, Heidenreich F, Tackenberg B, Weber-Schondorfer C, Myasth ABD (2010). Immunotherapy for myasthenia gravis and Lambert-Eaton Myasthenic Syndrome Part 2: intravenous Immunoglobulins and Apheresis Techniques. Aktuelle Neurologie.

[73] Herrlinger U, Weller M, Dichgans J, Melms A (2000). Association of primary central nervous system lymphoma with long-term azathioprine therapy for myasthenia gravis?. Ann Neurol.

[74] Higuchi O, Hamuro J, Motomura M, Yamanashi Y (2011). Autoantibodies to low-density lipoprotein receptor-related protein 4 in myasthenia gravis. Ann Neurol.

[75] Hill M, Beeson D, Moss P, Jacobson L, Bond A, Corlett L, Newsom-Davis J, Vincent A, Willcox N (1999). Early-onset myasthenia gravis: a recurring T-cell epitope in the adult-specific acetylcholine receptor epsilon subunit presented by the susceptibility allele HLA-DR52a. Ann Neurol.

[76] Hilton-Jones D (2007). When the patient fails to respond to treatment: myasthenia gravis. Pract Neurol.

[77] Hoch W, McConville J, Helms S, Newsom-Davis J, Melms A, Vincent A (2001). Auto-antibodies to the receptor tyrosine kinase MuSK in patients with myasthenia gravis without acetylcholine receptor antibodies. Nat Med.

[78] Hoeltzenbein M, Weber-Schoendorfer C, Borisch C, Allignol A, Meister R, Schaefer C (2012). Pregnancy outcome after paternal exposure to azathioprine/6-mercaptopurine. Reprod Toxicol.

[79] Hoffacker V, Schultz A, Tiesinga JJ, Gold R, Schalke B, Nix W, Kiefer R, Muller-Hermelink HK, Marx A (2000). Thymomas alter the T-cell subset composition in the blood: a potential mechanism for thymoma-associated autoimmune disease. Blood.

[80] Hohlfeld R, Goebels N, Engel AG (1993). Cellular mechanisms in inflammatory myopathies. Baillieres Clin Neurol.

[81] Hohlfeld R, Michels M, Heininger K, Besinger U, Toyka KV (1988). Azathioprine toxicity during long-term immunosuppression of generalized myasthenia gravis. Neurology.

[82] Hohlfeld R, Toyka KV, Besinger UA, Gerhold B, Heininger K (1985). Myasthenia gravis: reactivation of clinical disease and of autoimmune factors after discontinuation of long-term azathioprine. Ann Neurol.

[83] Howard JF, Barohn RJ, Cutter GR, Freimer M, Juel VC, Mozaffar T, Mellion ML, Benatar MG, Farrugia ME, Wang JJ, Malhotra SS, Kissel JT (2013). A randomized, double-blind, placebo-controlled phase II study of eculizumab in patients with refractory generalized myasthenia gravis. Muscle Nerve.

[84] Huang H, Benoist C, Mathis D (2010). Rituximab specifically depletes short-lived autoreactive plasma cells in a mouse model of inflammatory arthritis. Proc Natl Acad Sci USA.

[85] Huijbers MG, Zhang W, Klooster R, Niks EH, Friese MB, Straasheijm KR, Thijssen PE, Vrolijk H, Plomp JJ, Vogels P, Losen M, Van der Maarel SM, Burden SJ, Verschuuren JJ (2013). MuSK IgG4 autoantibodies cause myasthenia gravis by inhibiting binding between MuSK and Lrp4. Proc Natl Acad Sci USA.

[86] Imbach P, Barandun S, d’Apuzzo V, Baumgartner C, Hirt A, Morell A, Rossi E, Schoni M, Vest M, Wagner HP (1981). High-dose intravenous gammaglobulin for idiopathic thrombocytopenic purpura in childhood. Lancet.

[87] Iorio R, Damato V, Alboini PE, Evoli A (2015). Efficacy and safety of rituximab for myasthenia gravis: a systematic review and meta-analysis. J Neurol.

[88] Jaretzki A, Penn AS, Younger DS, Wolff M, Olarte MR, Lovelace RE, Rowland LP (1988). “Maximal” thymectomy for myasthenia gravis. Results. J Thorac Cardiovasc Surg.

[89] Kerty E, Elsais A, Argov Z, Evoli A, Gilhus NE (2014). EFNS/ENS Guidelines for the treatment of ocular myasthenia. Eur J Neurol.

[90] Kirchner T, Schalke B, Melms A, von Kugelgen T, Muller-Hermelink HK (1986). Immunohistological patterns of non-neoplastic changes in the thymus in Myasthenia gravis. Virchows Arch B Cell Pathol Incl Mol Pathol.

[91] Kisand K, Lilic D, Casanova JL, Peterson P, Meager A, Willcox N (2011). Mucocutaneous candidiasis and autoimmunity against cytokines in APECED and thymoma patients: clinical and pathogenetic implications. Eur J Immunol.

[92] Klooster R, Plomp JJ, Huijbers MG, Niks EH, Straasheijm KR, Detmers FJ, Hermans PW, Sleijpen K, Verrips A, Losen M, Martinez-Martinez P, De Baets MH, van der Maarel SM, Verschuuren JJ (2012). Muscle-specific kinase myasthenia gravis IgG4 autoantibodies cause severe neuromuscular junction dysfunction in mice. Brain.

[93] Kohler W, Bucka C, Klingel R (2011). A randomized and controlled study comparing immunoadsorption and plasma exchange in myasthenic crisis. J Clin Apher.

[94] Konishi T, Yoshiyama Y, Takamori M, Saida T (2005). Long-term treatment of generalised myasthenia gravis with FK506 (tacrolimus). J Neurol Neurosurg Psychiatry.

[95] Koziolek MJ, Kitze B, Muhlhausen J, Muller GA (2013). Immunoadsorption in steroid-refractory multiple sclerosis. Atheroscler Suppl.

[96] Kupersmith MJ (2009). Ocular myasthenia gravis: treatment successes and failures in patients with long-term follow-up. J Neurol.

[97] Lauriola L, Ranelletti F, Maggiano N, Guerriero M, Punzi C, Marsili F, Bartoccioni E, Evoli A (2005). Thymus changes in anti-MuSK-positive and -negative myasthenia gravis. Neurology.

[98] Leite MI, Jacob S, Viegas S, Cossins J, Clover L, Morgan BP, Beeson D, Willcox N, Vincent A (2008). IgG1 antibodies to acetylcholine receptors in ‘seronegative’ myasthenia gravis. Brain.

[99] Leite MI, Strobel P, Jones M, Micklem K, Moritz R, Gold R, Niks EH, Berrih-Aknin S, Scaravilli F, Canelhas A, Marx A, Newsom-Davis J, Willcox N, Vincent A (2005). Fewer thymic changes in MuSK antibody-positive than in MuSK antibody-negative MG. Ann Neurol.

[100] Lin PT, Martin BA, Weinacker AB, So YT (2006). High-dose cyclophosphamide in refractory myasthenia gravis with MuSK antibodies. Muscle Nerve.

[101] Lindberg C, Andersen O, Lefvert AK (1998). Treatment of myasthenia gravis with methylprednisolone pulse: a double blind study. Acta Neurol Scand.

[102] Luo J, Lindstrom J (2012). Myasthenogenicity of the main immunogenic region and endogenous muscle nicotinic acetylcholine receptors. Autoimmunity.

[103] Maclennan CA, Vincent A, Marx A, Willcox N, Gilhus NE, Newsom-Davis J, Beeson D (2008). Preferential expression of AChR epsilon-subunit in thymomas from patients with myasthenia gravis. J Neuroimmunol.

[104] Maniaol AH, Elsais A, Lorentzen AR, Owe JF, Viken MK, Saether H, Flam ST, Brathen G, Kampman MT, Midgard R, Christensen M, Rognerud A, Kerty E, Gilhus NE, Tallaksen CM, Lie BA, Harbo HF (2012). Late onset myasthenia gravis is associated with HLA DRB1*15:01 in the Norwegian population. PLoS One.

[105] Mantegazza R, Antozzi C, Peluchetti D, Sghirlanzoni A, Cornelio F (1988). Azathioprine as a single drug or in combination with steroids in the treatment of myasthenia gravis. J Neurol.

[106] Marx A, Pfister F, Schalke B, Saruhan-Direskeneli G, Melms A, Strobel P (2013). The different roles of the thymus in the pathogenesis of the various myasthenia gravis subtypes. Autoimmun Rev.

[107] Marx A, Wilisch A, Schultz A, Greiner A, Magi B, Pallini V, Schalke B, Toyka K, Nix W, Kirchner T, Muller-Hermelink HK (1996). Expression of neurofilaments and of a titin epitope in thymic epithelial tumors. Implications for the pathogenesis of myasthenia gravis. Am J Pathol.

[108] Marx A, Willcox N, Leite MI, Chuang WY, Schalke B, Nix W, Strobel P (2010). Thymoma and paraneoplastic myasthenia gravis. Autoimmunity.

[109] Masuda T, Motomura M, Utsugisawa K, Nagane Y, Nakata R, Tokuda M, Fukuda T, Yoshimura T, Tsujihata M, Kawakami A (2012). Antibodies against the main immunogenic region of the acetylcholine receptor correlate with disease severity in myasthenia gravis. J Neurol Neurosurg Psychiatry.

[110] McConville J, Farrugia ME, Beeson D, Kishore U, Metcalfe R, Newsom-Davis J, Vincent A (2004). Detection and characterization of MuSK antibodies in seronegative myasthenia gravis. Ann Neurol.

[111] Mehndiratta MM, Pandey S, Kuntzer T (2011) Acetylcholinesterase inhibitor treatment for myasthenia gravis. Cochrane Database Syst Rev CD006986 10.1002/14651858.CD006986.pub2 21328290

[112] Mertens HG, Balzereit F, Leipert M (1969). The treatment of severe myasthenia gravis with immunosuppressive agents. Eur Neurol.

[113] Meyer DM, Herbert MA, Sobhani NC, Tavakolian P, Duncan A, Bruns M, Korngut K, Williams J, Prince SL, Huber L, Wolfe GI, Mack MJ (2009). Comparative clinical outcomes of thymectomy for myasthenia gravis performed by extended transsternal and minimally invasive approaches. Ann Thorac Surg.

[114] Michels M, Hohlfeld R, Hartung HP, Heininger K, Besinger UA, Toyka KV (1988). Myasthenia gravis: discontinuation of long-term azathioprine. Ann Neurol.

[115] Minami N, Fujiki N, Doi S, Shima K, Niino M, Kikuchi S, Sasaki H (2011). Five-year follow-up with low-dose tacrolimus in patients with myasthenia gravis. J Neurol Sci.

[116] Muscle Study G (2008). A trial of mycophenolate mofetil with prednisone as initial immunotherapy in myasthenia gravis. Neurology.

[117] Nagaishi A, Yukitake M, Kuroda Y (2008). Long-term treatment of steroid-dependent myasthenia gravis patients with low-dose tacrolimus. Intern Med.

[118] Nagane Y, Utsugisawa K, Obara D, Kondoh R, Terayama Y (2005). Efficacy of low-dose FK506 in the treatment of myasthenia gravis–a randomized pilot study. Eur Neurol.

[119] Newsom-Davis J, Vincent A, Wilson SG, Ward CD, Pinching AJ, Hawkey C (1978). Plasmapheresis for myasthenia gravis. N Engl J Med.

[120] Nissenson AR (1977). Reduction of IgG levels in myasthenia. N Engl J Med.

[121] Novellino L, Longoni M, Spinelli L, Andretta M, Cozzi M, Faillace G, Vitellaro M, De Benedetti D, Pezzuoli G (1994). “Extended” thymectomy, without sternotomy, performed by cervicotomy and thoracoscopic technique in the treatment of myasthenia gravis. Int Surg.

[122] O’Donovan P, Perrett CM, Zhang X, Montaner B, Xu YZ, Harwood CA, McGregor JM, Walker SL, Hanaoka F, Karran P (2005). Azathioprine and UVA light generate mutagenic oxidative DNA damage. Science.

[123] Palace J, Newsom-Davis J, Lecky B (1998). A randomized double-blind trial of prednisolone alone or with azathioprine in myasthenia gravis. Myasthenia Gravis Study Group. Neurology.

[124] Pascuzzi RM, Coslett HB, Johns TR (1984). Long-term corticosteroid treatment of myasthenia gravis: report of 116 patients. Ann Neurol.

[125] Pevzner A, Schoser B, Peters K, Cosma NC, Karakatsani A, Schalke B, Melms A, Kroger S (2012). Anti-LRP4 autoantibodies in AChR- and MuSK-antibody-negative myasthenia gravis. J Neurol.

[126] Pharmaceuticals A (2013) Safety and efficacy of eculizumab in refractory generalized myasthenia gravis (REGAIN study). ClinicalTrialsgov web-page NCT01997229

[127] Pinching AJ, Peters DK (1976). Remission of myasthenia gravis following plasma-exchange. Lancet.

[128] Poea-Guyon S, Christadoss P, Le Panse R, Guyon T, De Baets M, Wakkach A, Bidault J, Tzartos S, Berrih-Aknin S (2005). Effects of cytokines on acetylcholine receptor expression: implications for myasthenia gravis. J Immunol.

[129] Pompeo E, Tacconi F, Massa R, Mineo D, Nahmias S, Mineo TC (2009). Long-term outcome of thoracoscopic extended thymectomy for nonthymomatous myasthenia gravis. Eur J Cardiothorac Surg.

[130] Ponseti JM, Azem J, Fort JM, Lopez-Cano M, Vilallonga R, Buera M, Cervera C, Armengol M (2005). Long-term results of tacrolimus in cyclosporine- and prednisone-dependent myasthenia gravis. Neurology.

[131] Ponseti JM, Gamez J, Azem J, Lopez-Cano M, Vilallonga R, Armengol M (2008). Tacrolimus for myasthenia gravis: a clinical study of 212 patients. Ann NY Acad Sci.

[132] Punga AR, Flink R, Askmark H, Stalberg EV (2006). Cholinergic neuromuscular hyperactivity in patients with myasthenia gravis seropositive for MuSK antibody. Muscle Nerve.

[133] Qureshi AI, Choudhry MA, Akbar MS, Mohammad Y, Chua HC, Yahia AM, Ulatowski JA, Krendel DA, Leshner RT (1999). Plasma exchange versus intravenous immunoglobulin treatment in myasthenic crisis. Neurology.

[134] Renton AE, Pliner HA, Provenzano C, Evoli A, Ricciardi R, Nalls MA, Marangi G, Abramzon Y, Arepalli S, Chong S, Hernandez DG, Johnson JO, Bartoccioni E, Scuderi F, Maestri M, Gibbs JR, Errichiello E, Chio A, Restagno G, Sabatelli M, Macek M, Scholz SW, Corse A, Chaudhry V, Benatar M, Barohn RJ, McVey A, Pasnoor M, Dimachkie MM, Rowin J, Kissel J, Freimer M, Kaminski HJ, Sanders DB, Lipscomb B, Massey JM, Chopra M, Howard JF, Koopman WJ, Nicolle MW, Pascuzzi RM, Pestronk A, Wulf C, Florence J, Blackmore D, Soloway A, Siddiqi Z, Muppidi S, Wolfe G, Richman D, Mezei MM, Jiwa T, Oger J, Drachman DB, Traynor BJ (2015). A genome-wide association study of myasthenia gravis. JAMA Neurol.

[135] Robertson NP, Deans J, Compston DA (1998). Myasthenia gravis: a population based epidemiological study in Cambridgeshire, England. J Neurol Neurosurg Psychiatry.

[136] Rodriguez M, Gomez MR, Howard FM, Taylor WF (1983). Myasthenia gravis in children: long-term follow-up. Ann Neurol.

[137] Romi F, Aarli JA, Gilhus NE (2007). Myasthenia gravis patients with ryanodine receptor antibodies have distinctive clinical features. Eur J Neurol.

[138] Romi F, Bo L, Skeie GO, Myking A, Aarli JA, Gilhus NE (2002). Titin and ryanodine receptor epitopes are expressed in cortical thymoma along with costimulatory molecules. J Neuroimmunol.

[139] Roxanis I, Micklem K, McConville J, Newsom-Davis J, Willcox N (2002). Thymic myoid cells and germinal center formation in myasthenia gravis; possible roles in pathogenesis. J Neuroimmunol.

[140] Ruckert JC, Gellert K, Muller JM (1999). Operative technique for thoracoscopic thymectomy. Surg Endosc.

[141] Sabbagh MN, Garza JS, Patten B (1995). Thoracoscopic thymectomy in patients with myasthenia gravis. Muscle Nerve.

[142] Samtleben W, Besinger UA, Toyka KV, Fateh-Moghadam A, Brehm G, Gurland HJ (1980). Plasma-separation in myasthenia gravis: a new method of rapid plasma exchange. Klin Wochenschr.

[143] Sanders DB, El-Salem K, Massey JM, McConville J, Vincent A (2003). Clinical aspects of MuSK antibody positive seronegative MG. Neurology.

[144] Sanders DB, Hart IK, Mantegazza R, Shukla SS, Siddiqi ZA, De Baets MH, Melms A, Nicolle MW, Solomons N, Richman DP (2008). An international, phase III, randomized trial of mycophenolate mofetil in myasthenia gravis. Neurology.

[145] Sanders DB, Howard JF, Johns TR (1979). Single-fiber electromyography in myasthenia gravis. Neurology.

[146] Sanes JR, Lichtman JW (2001). Induction, assembly, maturation and maintenance of a postsynaptic apparatus. Nat Rev Neurosci.

[147] Savino W, Manganella G, Verley JM, Wolff A, Berrih S, Levasseur P, Binet JP, Dardenne M, Bach JF (1985). Thymoma epithelial cells secrete thymic hormone but do not express class II antigens of the major histocompatibility complex. J Clin Invest.

[148] Schluep M, Willcox N, Vincent A, Dhoot GK, Newsom-Davis J (1987). Acetylcholine receptors in human thymic myoid cells in situ: an immunohistological study. Ann Neurol.

[149] Schneider-Gold C, Gajdos P, Toyka KV, Hohlfeld RR (2005) Corticosteroids for myasthenia gravis. Cochrane Database Syst Rev:CD002828 10.1002/14651858.CD002828.pub2 PMC8406927 15846640

[150] Schroder A, Linker RA, Gold R (2009). Plasmapheresis for neurological disorders. Expert Rev Neurother.

[151] Seldin MF, Alkhairy OK, Lee AT, Lamb JA, Sussman J, Pirskanen-Matell R, Piehl F, Verschuuren JJ, Kostera-Pruszczyk A, Szczudlik P, McKee D, Maniaol AH, Harbo HF, Lie BA, Melms A, Garchon HJ, Willcox N, Gregersen PK, Hammarstrom L (2015) Genome-wide Association Study of Late-Onset Myasthenia Gravis: Confirmation of TNFRSF11A, and Identification of ZBTB10 and Three Distinct HLA Associations. Mol Med 10.2119/molmed.2015.00232 PMC4749491 26562150

[152] Seybold ME (1998). Thymectomy in childhood myasthenia gravis. Ann N Y Acad Sci.

[153] Seybold ME, Drachman DB (1974). Gradually increasing doses of prednisone in myasthenia gravis. Reducing the hazards of treatment. N Engl J Med.

[154] Shen C, Lu Y, Zhang B, Figueiredo D, Bean J, Jung J, Wu H, Barik A, Yin DM, Xiong WC, Mei L (2013). Antibodies against low-density lipoprotein receptor-related protein 4 induce myasthenia gravis. J Clin Invest.

[155] Shigemoto K, Kubo S, Maruyama N, Hato N, Yamada H, Jie C, Kobayashi N, Mominoki K, Abe Y, Ueda N, Matsuda S (2006). Induction of myasthenia by immunization against muscle-specific kinase. J Clin Invest.

[156] Shiraishi H, Motomura M, Yoshimura T, Fukudome T, Fukuda T, Nakao Y, Tsujihata M, Vincent A, Eguchi K (2005). Acetylcholine receptors loss and postsynaptic damage in MuSK antibody-positive myasthenia gravis. Ann Neurol.

[157] Siara J, Rudel R, Marx A (1991). Absence of acetylcholine-induced current in epithelial cells from thymus glands and thymomas of myasthenia gravis patients. Neurology.

[158] Skeie GO, Apostolski S, Evoli A, Gilhus NE, Illa I, Harms L, Hilton-Jones D, Melms A, Verschuuren J, Horge HW, European Federation of Neurological S (2010). Guidelines for treatment of autoimmune neuromuscular transmission disorders. Eur J Neurol.

[159] Slesak G, Melms A, Fea Gerneth, Richman DP (1998). Late-onset myasthenia gravis– follow-up of 113 patients diagnosed after age 60. Myasthenia Gravis and Related Diseases: Disorders of the Neuromuscular Junction.

[160] Sommer N, Sigg B, Melms A, Weller M, Schepelmann K, Herzau V, Dichgans J (1997). Ocular myasthenia gravis: response to long-term immunosuppressive treatment. J Neurol Neurosurg Psychiatry.

[161] Stangel M, Gold R (2011). Administration of intravenous immunoglobulins in neurology. An evidence-based consensus: update 2010. Nervenarzt.

[162] Strobel P, Bauer A, Puppe B, Kraushaar T, Krein A, Toyka K, Gold R, Semik M, Kiefer R, Nix W, Schalke B, Muller-Hermelink HK, Marx A (2004). Tumor recurrence and survival in patients treated for thymomas and thymic squamous cell carcinomas: a retrospective analysis. J Clin Oncol.

[163] Strobel P, Chuang WY, Chuvpilo S, Zettl A, Katzenberger T, Kalbacher H, Rieckmann P, Nix W, Schalke B, Gold R, Muller-Hermelink HK, Peterson P, Marx A (2008). Common cellular and diverse genetic basis of thymoma-associated myasthenia gravis: role of MHC class II and AIRE genes and genetic polymorphisms. Ann NY Acad Sci.

[164] Strobel P, Helmreich M, Menioudakis G, Lewin SR, Rudiger T, Bauer A, Hoffacker V, Gold R, Nix W, Schalke B, Elert O, Semik M, Muller-Hermelink HK, Marx A (2002). Paraneoplastic myasthenia gravis correlates with generation of mature naive CD4(+) T cells in thymomas. Blood.

[165] Strobel P, Moritz R, Leite MI, Willcox N, Chuang WY, Gold R, Nix W, Schalke B, Kiefer R, Muller-Hermelink HK, Jaretzki Iii A, Newsom-Davis J, Marx A (2008). The ageing and myasthenic thymus: a morphometric study validating a standard procedure in the histological workup of thymic specimens. J Neuroimmunol.

[166] Strobel P, Murumagi A, Klein R, Luster M, Lahti M, Krohn K, Schalke B, Nix W, Gold R, Rieckmann P, Toyka K, Burek C, Rosenwald A, Muller-Hermelink HK, Pujoll-Borrell R, Meager A, Willcox N, Peterson P, Marx A (2007). Deficiency of the autoimmune regulator AIRE in thymomas is insufficient to elicit autoimmune polyendocrinopathy syndrome type 1 (APS-1). J Pathol.

[167] Strobel P, Rosenwald A, Beyersdorf N, Kerkau T, Elert O, Murumagi A, Sillanpaa N, Peterson P, Hummel V, Rieckmann P, Burek C, Schalke B, Nix W, Kiefer R, Muller-Hermelink HK, Marx A (2004). Selective loss of regulatory T cells in thymomas. Ann Neurol.

[168] Strober J, Cowan MJ, Horn BN (2009). Allogeneic hematopoietic cell transplantation for refractory myasthenia gravis. Arch Neurol.

[169] Tackenberg B, Hemmer B, Oertel WH, Sommer N (2001). Immunosuppressive treatment of ocular myasthenia gravis. BioDrugs.

[170] Tackenberg B, Nitschke M, Willcox N, Ziegler A, Nessler S, Schumm F, Oertel WH, Hemmer B, Sommer N (2003). CD45 isoform expression in autoimmune myasthenia gravis. Autoimmunity.

[171] Tackenberg B, Schlegel K, Happel M, Eienbroker C, Gellert K, Oertel WH, Meager A, Willcox N, Sommer N (2009). Expanded TCR Vbeta subsets of CD8(+) T-cells in late-onset myasthenia gravis: novel parallels with thymoma patients. J Neuroimmunol.

[172] Tada M, Shimohata T, Tada M, Oyake M, Igarashi S, Onodera O, Naruse S, Tanaka K, Tsuji S, Nishizawa M (2006). Long-term therapeutic efficacy and safety of low-dose tacrolimus (FK506) for myasthenia gravis. J Neurol Sci.

[173] Thiruppathi M, Rowin J, Ganesh B, Sheng JR, Prabhakar BS, Meriggioli MN (2012). Impaired regulatory function in circulating CD4(+)CD25(high)CD127(low/−) T cells in patients with myasthenia gravis. Clin Immunol.

[174] Tindall RS, Phillips JT, Rollins JA, Wells L, Hall K (1993). A clinical therapeutic trial of cyclosporine in myasthenia gravis. Ann NY Acad Sci.

[175] Toyka KV, Brachman DB, Pestronk A, Kao I (1975). Myasthenia gravis: passive transfer from man to mouse. Science.

[176] Toyka KV, Drachman DB, Griffin DE, Pestronk A, Winkelstein JA, Fishbeck KH, Kao I (1977). Myasthenia gravis. Study of humoral immune mechanisms by passive transfer to mice. N Engl J Med.

[177] Tracy MM, McRae W, Millichap JG (2009). Graded response to thymectomy in children with myasthenia gravis. J Child Neurol.

[178] Travis WD, Brambilla E, Burke AP, Marx A, Nicholson AG (2015) WHO classification of tumours of the lung, pleura, thymus and heart. IARC Press 4th ed 10.1097/JTO.0000000000000663 26291007

[179] Trontelj JV, Stalberg E (1995). Single fiber electromyography in studies of neuromuscular function. Adv Exp Med Biol.

[180] Tumani H (2008). Corticosteroids and plasma exchange in multiple sclerosis. J Neurol.

[181] Tzartos SJ, Lindstrom JM (1980). Monoclonal antibodies used to probe acetylcholine receptor structure: localization of the main immunogenic region and detection of similarities between subunits. Proc Natl Acad Sci USA.

[182] Van de Velde RL, Friedman NB (1970). Thymic myoid cells and myasthenia gravis. Am J Pathol.

[183] Vernino S, Salomao DR, Habermann TM, O’Neill BP (2005). Primary CNS lymphoma complicating treatment of myasthenia gravis with mycophenolate mofetil. Neurology.

[184] Vincent A, Jacobson L, Shillito P (1994). Response to human acetylcholine receptor alpha 138–199: determinant spreading initiates autoimmunity to self-antigen in rabbits. Immunol Lett.

[185] Wakkach A, Poea S, Chastre E, Gespach C, Lecerf F, De La Porte S, Tzartos S, Coulombe A, Berrih-Aknin S (1999). Establishment of a human thymic myoid cell line. Phenotypic and functional characteristics. Am J Pathol.

[186] Willcox N, Leite MI, Kadota Y, Jones M, Meager A, Subrahmanyam P, Dasgupta B, Morgan BP, Vincent A (2008). Autoimmunizing mechanisms in thymoma and thymus. Ann NY Acad Sci.

[187] Willcox N, Schluep M, Ritter MA, Schuurman HJ, Newsom-Davis J, Christensson B (1987). Myasthenic and nonmyasthenic thymoma. An expansion of a minor cortical epithelial cell subset?. Am J Pathol.

[188] Witte AS, Cornblath DR, Parry GJ, Lisak RP, Schatz NJ (1984). Azathioprine in the treatment of myasthenia gravis. Ann Neurol.

[189] Witte AS, Cornblath DR, Schatz NJ, Lisak RP (1986). Monitoring azathioprine therapy in myasthenia gravis. Neurology.

[190] Wolfe GI, Kaminski HJ, Jaretzki A, Swan A, Newsom-Davis J (2003). Development of a thymectomy trial in nonthymomatous myasthenia gravis patients receiving immunosuppressive therapy. Ann NY Acad Sci.

[191] Yeh JH, Chiu HC (2000). Comparison between double-filtration plasmapheresis and immunoadsorption plasmapheresis in the treatment of patients with myasthenia gravis. J Neurol.

[192] Yim AP, Kay RL, Ho JK (1995). Video-assisted thoracoscopic thymectomy for myasthenia gravis. Chest.

[193] Yoshikawa H, Kiuchi T, Saida T, Takamori M (2011). Randomised, double-blind, placebo-controlled study of tacrolimus in myasthenia gravis. J Neurol Neurosurg Psychiatry.

[194] Zeitler H, Ulrich-Merzenich G, Hoffmann L, Kornblum C, Schmidt S, Vetter H, Walger P (2006). Long-term effects of a multimodal approach including immunoadsorption for the treatment of myasthenic crisis. Artif Organs.

[195] Zhang B, Shen C, Bealmear B, Ragheb S, Xiong WC, Lewis RA, Lisak RP, Mei L (2014). Autoantibodies to agrin in myasthenia gravis patients. PLoS One.

[196] Zhang B, Tzartos JS, Belimezi M, Ragheb S, Bealmear B, Lewis RA, Xiong WC, Lisak RP, Tzartos SJ, Mei L (2012). Autoantibodies to lipoprotein-related protein 4 in patients with double-seronegative myasthenia gravis. Arch Neurol.

[197] Zhou L, McConville J, Chaudhry V, Adams RN, Skolasky RL, Vincent A, Drachman DB (2004). Clinical comparison of muscle-specific tyrosine kinase (MuSK) antibody-positive and -negative myasthenic patients. Muscle Nerve.

[198] Zinman L, Ng E, Bril V (2007). IV immunoglobulin in patients with myasthenia gravis: a randomized controlled trial. Neurology.

[199] Zisimopoulou P, Evangelakou P, Tzartos J, Lazaridis K, Zouvelou V, Mantegazza R, Antozzi C, Andreetta F, Evoli A, Deymeer F, Saruhan-Direskeneli G, Durmus H, Brenner T, Vaknin A, Berrih-Aknin S, Frenkian Cuvelier M, Stojkovic T, DeBaets M, Losen M, Martinez-Martinez P, Kleopa KA, Zamba-Papanicolaou E, Kyriakides T, Kostera-Pruszczyk A, Szczudlik P, Szyluk B, Lavrnic D, Basta I, Peric S, Tallaksen C, Maniaol A, Tzartos SJ (2014). A comprehensive analysis of the epidemiology and clinical characteristics of anti-LRP4 in myasthenia gravis. J Autoimmun.

